# Red-tailed hawk algorithm for numerical optimization and real-world problems

**DOI:** 10.1038/s41598-023-38778-3

**Published:** 2023-08-09

**Authors:** Seydali Ferahtia, Azeddine Houari, Hegazy Rezk, Ali Djerioui, Mohamed Machmoum, Saad Motahhir, Mourad Ait-Ahmed

**Affiliations:** 1 Institut de Recherche en Énergie Électrique de Nantes Atlantique, IREENA, Nantes University, Saint-Nazaire, France; 2 Laboratoire de Génie Electrique, Dept. of Electrical Engineering, University of M’sila, M’sila, Algeria; 3 College of Engineering at Wadi Addawaser, Prince Sattam Bin Abdulaziz University, Al-Kharj, Saudi Arabia; 4 ENSA, University of Sidi Mohamed Ben Abdellah, Fez, Morocco

**Keywords:** Fuel cells, Electrical and electronic engineering

## Abstract

This study suggests a new nature-inspired metaheuristic optimization algorithm called the red-tailed hawk algorithm (RTH). As a predator, the red-tailed hawk has a hunting strategy from detecting the prey until the swoop stage. There are three stages during the hunting process. In the high soaring stage, the red-tailed hawk explores the search space and determines the area with the prey location. In the low soaring stage, the red-tailed moves inside the selected area around the prey to choose the best position for the hunt. Then, the red-tailed swings and hits its target in the stooping and swooping stages. The proposed algorithm mimics the prey-hunting method of the red-tailed hawk for solving real-world optimization problems. The performance of the proposed RTH algorithm has been evaluated on three classes of problems. The first class includes three specific kinds of optimization problems: 22 standard benchmark functions, including unimodal, multimodal, and fixed-dimensional multimodal functions, IEEE Congress on Evolutionary Computation 2020 (CEC2020), and IEEE CEC2022. The proposed algorithm is compared with eight recent algorithms to confirm its contribution to solving these problems. The considered algorithms are Farmland Fertility Optimizer (FO), African Vultures Optimization Algorithm (AVOA), Mountain Gazelle Optimizer (MGO), Gorilla Troops Optimizer (GTO), COOT algorithm, Hunger Games Search (HGS), Aquila Optimizer (AO), and Harris Hawks optimization (HHO). The results are compared regarding the accuracy, robustness, and convergence speed. The second class includes seven real-world engineering problems that will be considered to investigate the RTH performance compared to other published results profoundly. Finally, the proton exchange membrane fuel cell (PEMFC) extraction parameters will be performed to evaluate the algorithm with a complex problem. The proposed algorithm will be compared with several published papers to approve its performance. The ultimate results for each class confirm the ability of the proposed RTH algorithm to provide higher performance for most cases. For the first class, the RTH mostly got the optimal solutions for most functions with faster convergence speed. The RTH provided better performance for the second and third classes when resolving the real word engineering problems or extracting the PEMFC parameters.

## Introduction

Optimization algorithms have played a vital role in recent decades in assisting engineers and designers in finding improved solutions for saving time, money, and energy^1^. Numerical optimization methods often employ simple and widely used models^2^. Nevertheless, these algorithms require several gradient information to find superior solutions circling a specific point in a small space^3^. Furthermore, they are sensitive to starting points, mainly when the issues under consideration include multiple local solutions. Inappropriate initial point selection makes searching for the global optimum solution complex and inconsistent^4^. In recent years, many complicated optimization problems have evolved in various disciplines. These problems frequently involve several choices of optimization variables, complex nonlinear constraints, and objective functions^5^. These algorithms have been used for civil engineering^6^, electrical engineering, image processing^7^, medical and biological applications, and others.

As a result, existing numerical approaches cannot address these complicated problems promptly and precisely. On the other hand, nature inspiration can provide concepts to develop artificial intelligence algorithms to solve these complex problems.

Recently, various nature-inspired metaheuristic algorithms (MAs) have been developed. These MAs mimic the motions of live beings or natural events. These MAs have been elaborated and employed for solving many optimization issues as competitive alternative solvers^8^. However, these MAs have a common drawback; they frequently exhibit an extra sensitivity to adjusting user-defined parameters. Another disadvantage is that MAs may not always reach the global optimum solution^9^. MAs are divided into two types^8^: single solution-based and population-based. A single solution is processed during optimization in the single solution-based type. In contrast, in the population-based type, solutions are developed in each optimization iteration. Population-based MAs initiate the optimization process by producing a set of random individuals. Each of them represents a possible optimal solution. Iteratively, the population will be developed by substituting the actual population with a recently created one based on certain stochastic operators^10,11^.

Regardless of how diverse these algorithms are, they always have one common characteristic: the searching operations are divided into two phases: exploration and exploitation^12^. As a result, during the early stages of the search process, a well-designed optimizer's exploratory behaviors must have an enriched-enough random character to distribute more random solutions effectively^13^. Hence, it enhanced diverse parts of the search space. After the exploration phase, the exploitation phase is carried out. The optimizer accelerates the search process in a narrow area instead of the whole search space by concentrating on the neighborhood of the best-obtained solutions. An efficient optimizer must strike an acceptable and precise balance between the exploration and exploitation phases. Otherwise, the risk of becoming locked in local optima (local solutions) and having immature convergence downsides grows. According to the No Free Lunc (NFL) theorem [36], all the proposed MAs show a comparable average performance when resolving all possible optimization problems. In other words, no algorithm can be considered a general best algorithm. As a result, the NFL theorem promotes creating and developing more new efficient optimizers. Technically, each MA employs unique evolution mechanisms. According to^14^, several fundamental concepts can be used to characterize them. Among these concepts:

- *Parallelism* is utilized in population-based algorithms such as SA and HA. Different individuals are sent out at once to complete a task, and the results are compared. According to the comparison, additional ideas are utilized to assess the population evolution or to create new individuals.
- *Acceptance* is utilized in three cases: 1. Admit temporary solutions that weaken objective function due to search space expansion, 2. Handling the cost function's constraints. There are two ways to cope with the constraints. The first way excludes any solutions if they match the start conditions. The second procedure is used if any solution can be allocated a numerical value. In this case, all solutions must participate, and the initial conditions may match infeasible solutions. 3. Adding restrictions to allowed solutions that improve the optimal solution by, at minimum, the limiting level. When comparing values produced from previous calculations, this strategy aids in avoiding numerical issues.
- *Elitism*: the best solution must be kept from one iteration to the next in repeating population-based algorithms. The elitism principle is utilized to accomplish this by retaining the individual who outlined the best solution and utilizing it as a reference for the following iteration or upgrading it if a better solution is located. The notion of elitism may also be applied to many individuals, directing an élite group of them to the next iteration.
- *Selection* is a probability-based method that generates new individuals from a pool of available ones. This approach may incorporate weights into the probabilistic selection process, in which random individuals are chosen to produce new ones.
- *Decay or Reinforcement*: Decay allows for more initial freedom, followed by incremental flexibility constraints. This approach is based on a decremental updating factor (< 1) used at each iteration. Reinforcement is employed similarly in some cases by applying an incremental factor higher (> 1).
- *Immunity* is gained through finding characteristics of solutions that lead to appropriate settings. Immunity prefers solutions with characteristics similar to those attributes.
- *Self-Adaptation* is a process that allows the parameters of algorithms to be updated in response to the progress of optimization.

A novel high-efficiency SA optimization algorithm is suggested in this study to compete with existing optimizers. The basic concept of the suggested optimizer is based on the hunting skills of Red-tailed Hawks, one of the most intelligent birds of prey. The benefits of evolutionary and swarm approaches have been included in the RTH algorithm's design to outperform the existing optimization algorithms. The proposed RTH contains three stages: high soaring, low soaring, and stooping and swooping stages. The high soaring stage is quite similar to evolutionary approaches' search behavior. The RTH algorithm relays and gathers all search points, beginning with the original position and progressing to the best position. The second and third phases imitate SA's behavior while moving to the best position, using the information of the preceding site for each point.

Despite the availability of metaheuristic algorithms, there is a continuous need for developing and creating new algorithms that may provide better performance for such problems than the actual ones. The NFL theory confirms this need for new optimizers. To this end, this paper suggests this novel algorithm. The main contribution of this paper is to propose a novel nature-inspired metaheuristic optimization algorithm for better solving optimization issues. The proposed algorithm benefit from the unique hunting strategy of the red-tailed hawk and applies it to tackle various optimization problems. The suggested RTH algorithm's performance has been examined on three optimization problem classes. The first class contains three types of optimization functions:

- Twenty-three standard benchmark functions: unimodal, multimodal, and fixed-dimensional multimodal functions.
- IEEE Congress on Evolutionary Computation 2020 (CEC2020) with 15 and 20 search space dimensions.
- IEEE Congress on Evolutionary Computation 2022 (CEC2022) with 10 and 20 search space dimensions.

The proposed algorithm is compared with eight recent algorithms to confirm its contribution to solving the problems of this class. The considered algorithms in the comparison are Farmland fertility Optimizer (FO)^15^, African Vultures Optimization Algorithm (AVOA)^16^, Mountain Gazelle Optimizer (MGO)^17^, Artificial Gorilla Troops Optimizer (GTO)^18^, COOT algorithm^19^, Hunger Games Search (HGS)^20^, Aquila Optimizer (AO)^21^, and Harris Hawks optimization (HHO)^22^. The findings' accuracy (mean value), robustness (standard deviation), and convergence speed are compared. The second class consists of seven real-world engineering problems that will be thoroughly investigated compared to previously published solutions. These problems include the optimal design of an I-shaped beam, a three-bar truss Design, design of a tubular column, a piston lever, a corrugated design, and design tension/compression spring. The third class provides the parameters extraction for proton exchange membrane fuel cell (PEMFC), which will be used to assess the suggested algorithm with a difficult task. To validate its performance, the proposed algorithm will be compared to numerous published articles for three types of PRMFC: BSC 500W, NedStack PS6, and SR-12 500W.

The rest of this paper is organized as follows: Section “Related works” presents a related works part that shows and explains a set of related works. Section “Red-tailed hawk algorithm” reviews the hunting behavior of red-tailed, gives the inspiration source, and describes each stage of the proposed RTH algorithm. This section includes the mathematical model of the proposed algorithm. Section “Results and discussion” presents the outcomes of RTH in solving the considered problems. This paper ends with a conclusion in Section “Engineering optimization problems”.

## Related works

In recent decades, there has been a rise in the assessment and application of metaheuristic algorithms to tackle optimization problems. In the literature, the population-based MAs may be divided into four categories based on their inspiration^23,24^: evolutionary (EA), Physics-based (PA), Human-based (HA), and swarm-based MAs (SA). EAs imitate biological evolutionary processes, including recombination, mutation, and selection. The Genetic Algorithm (GA)^25,26^, Differential Evolution (DE)^27^, Biogeography-Based Optimizer (BBO)^28^, and Mind evolutionary algorithm optimization (MEDA)^29^ are the most well-known EAs. Physical phenomena-inspired algorithms are based on physical laws such as gravity, magnetic force, etc. Gravitational Search Algorithm (GSA)^30^, Gradient-based optimizer^31^, and Energy Valley Optimizer (EVO)^32^ are a few examples. Human-based MAs imitate some human activities and behaviors. Socio Evolution and Learning Optimization (SELO)^33^, Social Network Search (SNS)^34^, and Human Felicity Algorithm (HFA)^35^ are some examples of this category. Swarm-based MAs mimic the social behaviors of animals or organisms living in swarms, communities, or packs^36^. Particle Swarm Optimization (PSO)^37^, Salp Swarm Algorithm (SSA)^38^, and Jellyfish Search algorithm (JSA)^39^ are the most well-known MAs in this category. The swarm-based MAs have been getting more attention in the last years due to the availability of inspiration sources and their efficacy in resolving various optimization problems. New papers propose or review this category of optimization algorithms, such as Mountain Gazelle Optimizer (MGO)^17^, Advances in Spotted Hyena Optimizer^40^, Advances in Tree Seed Algorithm^41^, Advances in Sparrow Search Algorithm^42^, Butterfly Optimization Algorithm (BOA)^43^, Advanced Butterfly Optimization Algorithm (ABOA)^44^, Modified Butterfly Optimization Algorithm with Lagrange Interpolation (MBOALI)^45^, African Vultures Optimization Algorithm (AVOA)^16^, Whale optimization algorithm (WOA)^46^, Artificial Gorilla Troops Optimizer (GTO)^18^, COOT algorithm^19^, Weibull Flight based Moth Flame Optimization (WF-MFO)^47^, Hunger Games Search (HGS)^20^, Aquila Optimizer (AO)^21^ and so on. On the other hand, Quantum-inspired metaheuristic algorithms developed by combining Quantum Computing (QC) principles into metaheuristic algorithms are gaining more interest^48^. The performance of these algorithms is considerably enhanced by using the QC for boost exploration and exploitation and faster convergence.

## Red-tailed hawk algorithm

This part discusses the proposed RTH algorithm. The inspiration source and hunting strategy are discussed in the first subsection. Then, the mathematical model mimicking the red-tailed hawk's behavior is presented, and each stage of the algorithm is analyzed.

### Inspiration and behavior during hunting

The red-tailed hawk (*Buteo jamaicensis*) is a bird of prey that breeds over much of North America, from Alaska's interior and northern Canada to Panama and the West Indies. The red-tailed hawk lives in various environments and elevations, such as deserts, grasslands, forests, agricultural fields, and cities. The red-tailed hawk is a predatory, carnivorous eater. Almost every little animal they come upon may be seen as possible prey^49^. Small mammals, such as rodents, are their most common prey, although they also eat birds, fish, reptiles, invertebrates, and amphibians. Prey varies greatly depending on geographical and seasonal availability; however, rodents comprise 85 percent of a hawk's diet^50^.

**Figure 1:**
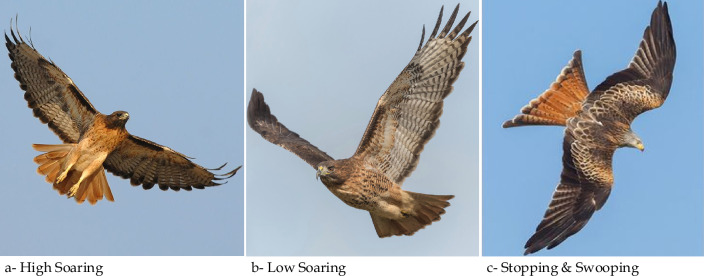
Behavior of red-tailed hawk during hunting.

The red-tailed hawk soars with its wings in a mild dihedral, flapping as little as possible to save energy. Unlike other hawks, the red-tails can fly for long distances thanks to this feature. Because soaring is the most efficient flying mode for these hawks, it is utilized more frequently^51^. It moves between 32 and 64 km/h (20–40 mph) when soaring or flapping its wings. The large wings allow the red-tailed to reach 190 km/h (120 mph) when plunging^52^. Red-tailed hawks can fly fast and powerfully while repeatedly diving at perceived threats during nest protection^53^. As illustrated in Fig. 1, the red-tailed has three types of flying:

- High soaring (Fig. 1a): It flies highly with its wings in a mild dihedral, flapping as little as possible to save energy to explore the selected area.
- Low soaring (Fig. 1b): after selecting the target position, the red-tailed fly with a low soaring in a spiral movement around the prey. This movement allows it to detect the best location and time to hit the target.
- Stooping and swooping (Fig. 1c): after selecting the best location and moment in the previous step, the red-tailed swooped its prey by stooping and raising its acceleration (from 32–64 to 190 km/h) in a curved direction.

### Mathematical model

The suggested red-tailed hawk (RTH) algorithm mimics the red-tailed hawk's hunting behavior. The actions taken at each hunt stage are presented and modeled. This algorithm includes three stages, high soaring, low soaring, and stooping and swooping.

1. High soaring: the red-tailed hawk will soar far into the sky, looking for the best location in terms of food availability. Figure 2 illustrates the behavior of the red-tailed hawks during the high soaring stage, and Eq. (1) represents the mathematical model of this stage :
  1 $$X(t) = X_{best} + (X_{mean} - X(t - 1)) \cdot Levy(\dim ) \cdot TF(t)$$
  where X(t) represents the red-tailed hawk position at the iteration *t*, *X*_*best*_ is the best-obtained position, *X*_*mean*_ is the positions' mean, Levy represents the levy flight distribution function that can be calculated according to Eq. (2), and TF(t) denotes the transition factor function that can be calculated according to Eq. (3).
  2 $$\begin{aligned} & Levy(\dim ) = s\frac{\mu \cdot \sigma }{{\left| \upsilon \right|^{{\beta^{ - 1} }} }} \\ & \sigma = \left( {\frac{{\Gamma (1 + \beta ) \cdot \sin \left( {{\raise0.7ex\hbox{${\pi \beta }$} \!\mathord{\left/ {\vphantom {{\pi \beta } 2}}\right.\kern-0pt} \!\lower0.7ex\hbox{$2$}}} \right)}}{{\Gamma ({\raise0.7ex\hbox{${1 + \beta }$} \!\mathord{\left/ {\vphantom {{1 + \beta } 2}}\right.\kern-0pt} \!\lower0.7ex\hbox{$2$}}) \cdot \beta \cdot 2^{{\left( {{\raise0.7ex\hbox{${1 - \beta }$} \!\mathord{\left/ {\vphantom {{1 - \beta } 2}}\right.\kern-0pt} \!\lower0.7ex\hbox{$2$}}} \right)}} }}} \right) \\ \end{aligned}$$
  where *s* is a constant (0.01), dim is the problem dimension, *β* is a constant (1.5), *u*, and *υ* are random numbers [0 to 1].
  3 $$TF(t) = 1 + \sin \left( {2.5 + \left( {{\raise0.7ex\hbox{$t$} \!\mathord{\left/ {\vphantom {t {T_{\max } }}}\right.\kern-0pt} \!\lower0.7ex\hbox{${T_{\max } }$}}} \right)} \right)$$
  where *T*_*max*_ represents the max number of iterations.

**Figure 2:**
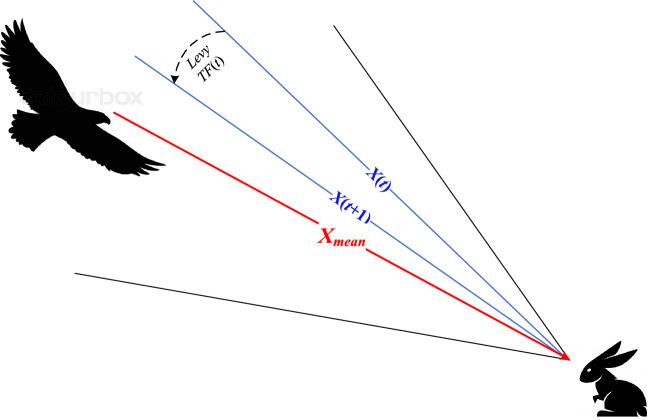
Behavior of red-tailed hawk during high soaring stage.
2. Low soaring: the hawk surrounds the prey by flying much lower to the ground in a spiral line. This stage is illustrated in Fig. 3, and its model can be expressed as follows:
  4 $$\begin{aligned} & X(t) = X_{best} + (x(t) + y(t)) \cdot {\text{StepSize}}(t) \\ & {\text{StepSize}}(t) = X(t) - X_{mean} \\ \end{aligned}$$
  where *x* and *y* denote direction coordinates which can be calculated as follows
  5 $$\begin{array}{*{20}c} {\left\{ \begin{gathered} x(t) = R(t) \cdot \sin \left( {\theta (t)} \right) \hfill \\ y(t) = R(t) \cdot \cos \left( {\theta (t)} \right) \hfill \\ \end{gathered} \right.} & {\left\{ \begin{gathered} R(t) = R_{0} \cdot \left( {r - {\raise0.7ex\hbox{$t$} \!\mathord{\left/ {\vphantom {t {T_{\max } }}}\right.\kern-0pt} \!\lower0.7ex\hbox{${T_{\max } }$}}} \right) \cdot rand \hfill \\ \theta (t) = A \cdot \left( {1 - {\raise0.7ex\hbox{$t$} \!\mathord{\left/ {\vphantom {t {T_{\max } }}}\right.\kern-0pt} \!\lower0.7ex\hbox{${T_{\max } }$}}} \right) \cdot rand \hfill \\ \end{gathered} \right.} \\ \end{array} \,\,\left\{ \begin{gathered} x(t) = {\raise0.7ex\hbox{${x(t)}$} \!\mathord{\left/ {\vphantom {{x(t)} {\max \left| {x(t)} \right|}}}\right.\kern-0pt} \!\lower0.7ex\hbox{${\max \left| {x(t)} \right|}$}} \hfill \\ y(t) = {\raise0.7ex\hbox{${y(t)}$} \!\mathord{\left/ {\vphantom {{y(t)} {\max \left| {y(t)} \right|}}}\right.\kern-0pt} \!\lower0.7ex\hbox{${\max \left| {y(t)} \right|}$}} \hfill \\ \end{gathered} \right.$$
  where *R*_*0*_ represents the initial value of the radius [0.5–3], *A* denotes the angel gain [5–15], *rand* is a random gain [0–1], and *r* is a control gain [1, 2]. These parameters help the hawk fly around the prey with spiral movements, as explained in Fig. 4.

**Figure 3:**
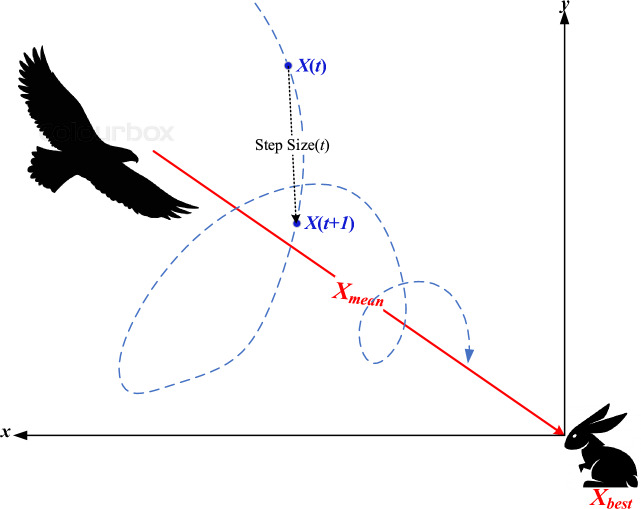
Behavior of red-tailed hawk during low soaring stage.

**Figure 4:**
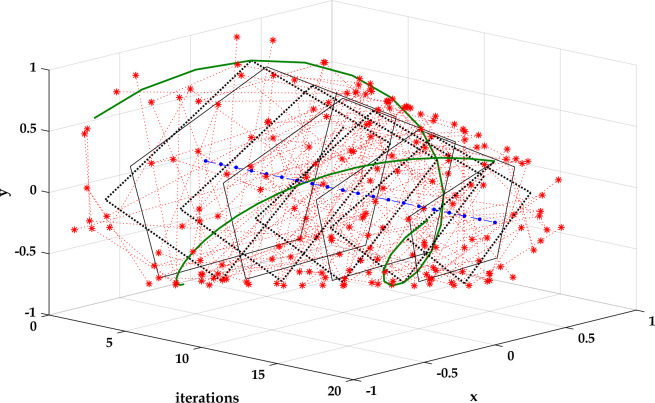
Evolution of the direction coordinates as a function of iterations.
3. Stooping and Swooping: In this stage, the hawk suddenly stoops and attacks the prey from the best-obtained position in the low soaring stage. Figure 2 explains the behavior of the red-tailed hawks during this stage. This stage can be modeled as follows:
  6 $$X(t) = \alpha (t) \cdot X_{best} + x(t) \cdot {\text{StepSize1}}(t) + y(t) \cdot {\text{StepSize2}}(t)$$
  where each step size can be calculated as follows
  7 $$\begin{aligned} & {\text{StepSize1}}(t) = X(t) - TF(t) \cdot X_{mean} \\ & {\text{StepSize2}}(t) = G(t) \cdot X(t) - TF(t) \cdot X_{best} \\ \end{aligned}$$
  where *α* and *G* are the acceleration and the gravity factors, respectively, they can be defined as follows:
  8 $$\begin{aligned} & \alpha (t) = \sin^{2} \left( {2.5 - {\raise0.7ex\hbox{$t$} \!\mathord{\left/ {\vphantom {t {T_{\max } }}}\right.\kern-0pt} \!\lower0.7ex\hbox{${T_{\max } }$}}} \right) \\ & G(t) = 2 \cdot \left( {1 - {\raise0.7ex\hbox{$t$} \!\mathord{\left/ {\vphantom {t {T_{\max } }}}\right.\kern-0pt} \!\lower0.7ex\hbox{${T_{\max } }$}}} \right) \\ \end{aligned}$$
  where *α* represents the hawk's acceleration that increases with the increase of t to enhance the convergence speed, and *G* is the gravity effect that decreases to reduce the exploitation diversity when the hawk is much near the prey. This phase is explained in Fig. 5.

**Figure 5:**
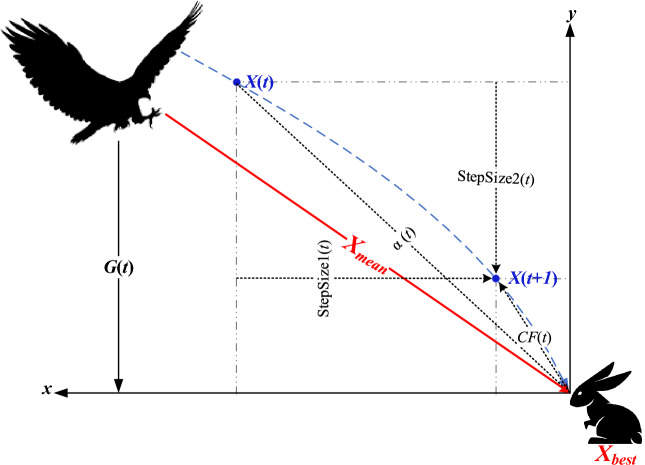
Behavior of red-tailed hawk during stooping and swooping stages.

**Figure 6:**
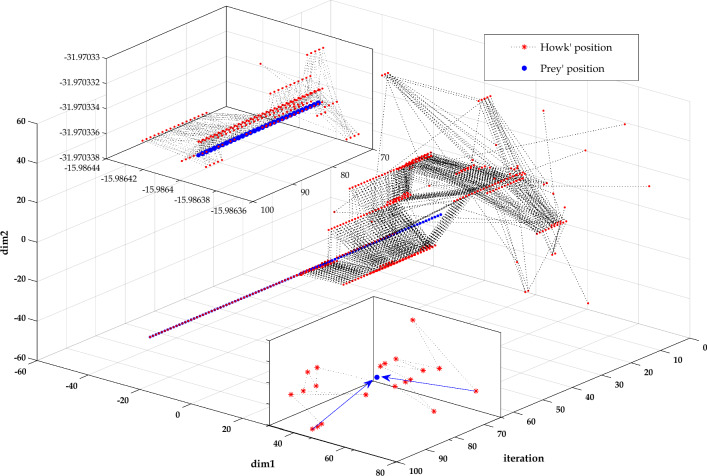
Agents' evolution as a function of the iterations for a 2-dimensional problem.

**Figure 7:**
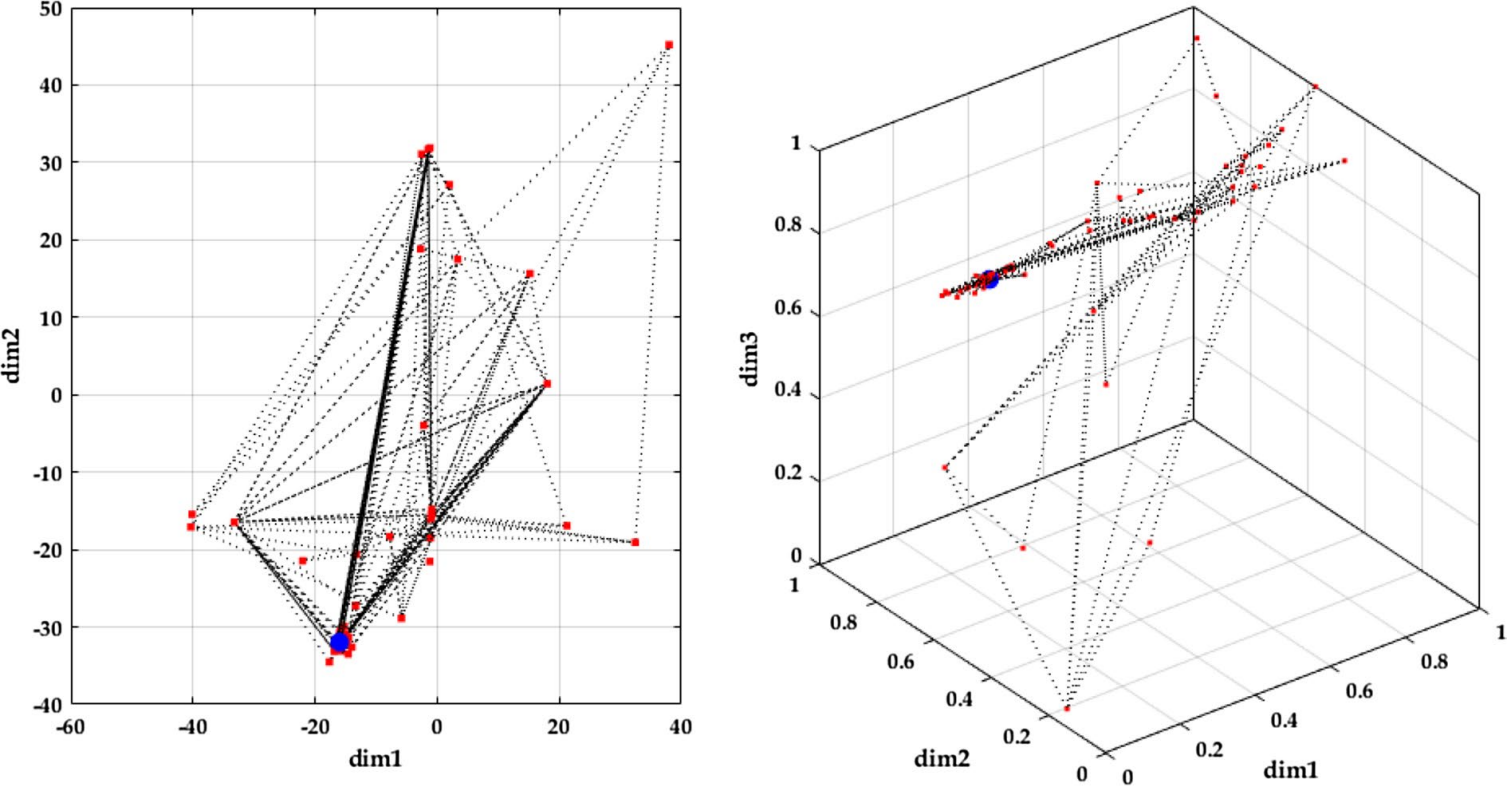
Agents' evolution for 2-dimensional and 3-dimensional problems.

Figure 6 presents the evolution of the solutions of a 2-dimensional problem as a function of the iterations. During the first iterations, the hawk explored the search space (exploration phase). It took significant steps to detect the prey position. The length of these steps decreases as the hawk is near the prey position. The hawk spent a small number of iterations in the exploration due to the hybrid evolutionary-swarm updating mechanism. It avoided attempting on the local minim thanks to the high soaring stage based on the Levy function. The low soaring enhances the convergence speed in the exploitation phase, where the stooping and the swooping stages strengthen the RTH algorithm's accuracy. Figure 7 presents the hunting behavior for 2-dimensional and 3-dimensional problems. The hawk explores the whole search space from these figures to avoid attempting local solutions. Due to its unique updating mechanism gets closer to the optimal solution without falling into local ones with a fast convergence rate.

To better understand the characteristics of the proposed RTH and its basics, the RTH concepts are presented in Table 1.

**Table 1:** The RTH concepts.

Concept	Projection on the algorithm
Classification according to inspiration type	Swarm-based
Classification according to evolution type	Population-based
Parallelism	The algorithm uses several positions and their mean value
Acceptance	Not used due to the low random behavior of the algorithm
Elitism	Used at each phase (*X*_*best*_)
Selection	The high soaring phase used this concept based on Levey's distribution
Decay	Used in *α* and *G* Eq. (8) to raise the exploration ability and converge toward the best solution
Reinforcement	Used in *TF* Eq. (3) to enhance the exploitation ability and avoid attempting in local optima
Immunity	Not used
Self-adaptation	Used in *TF* Eq. (3) and *α* and *G* Eq. (8), these parameters are updated according to the current iteration


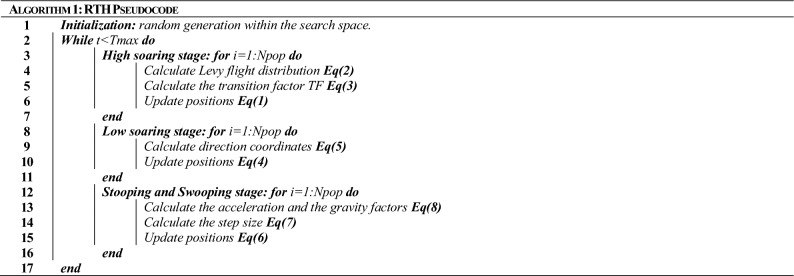


## Results and discussion

RTH's performance was evaluated using several test functions. Three classes of test functions are used in this study:

- Standard test functions^54^,
- CEC 2020 benchmark test functions^55^,
- CEC 2022 benchmark test functions^56^.

### Standard test functions

Unimodal, multimodal, and fixed-dimension multimodal test functions are used to evaluate the RTH performance^54^. Unimodal test functions (F1-F7) are used to put an algorithm's exploitation abilities to the test, whereas multimodal test functions (F8-F13) are used to experiment with the algorithm's exploration performance. The fixed dimension test functions (TF14-TF23) demonstrate the algorithm exploration capability^57^. These functions are provided in Table A1 (appendix). For all the tests, the population size for each algorithm has been set at 30 with max iterations of 1000.

This study ran RTH 30 times and reported the findings, including the average and standard deviation values of the best-so-far solutions obtained in each run to provide statistically significant results. The test is carried out for six other algorithms to underline the effectiveness and superiority of RTH to that of different methods: Particle Swarm Optimization (PSO)^37^ as the best-known optimization method, Salp Swarm Algorithm (SSA)^38^ as one of the most used algorithms, Hunger Games Search (HGS)^20^, COOT algorithm^19^, Artificial Eco-system Optimizer (AEO)^4^, and Aquila Optimizer (AO)^21^ as recently developed metaheuristics. Table 2 summarizes the parameter setting for the RTH algorithm, where the parameters are set by the try-and-error method. The other algorithms are based on their default parameters. Table 3 shows the results of RTH and the other used algorithms on these standard test functions using the best, average, and standard deviation (StD) values.

**Table 2:** The RTH paramters.

Parameter	Range	Cosen value
*A*	[5–25]	15
*R*_*0*_	[0.1–2]	0.5
*r*	[0.1–2]	1.5

**Table 3:** standard test functions results.

Func	Metric	MGO	FO	COOT	HGS	AO	HHO	GTO	AVAO	RTH
Unimodal functions										
F1	Best	1.21 × 10^−254^	1.46 × 10^−38^	9.63 × 10^−4^	**0**	**0**	1.95 × 10^−13^	**0**	**0**	**0**
	Mean	6.50 × 10^−240^	1.93 × 10^−35^	4.22 × 10^−3^	**0**	7.71 × 10^−200^	9.75 × 10^−9^	**0**	**0**	**0**
	StD	**0**	5.11 × 10^−35^	3.30 × 10^−3^	**0**	**0**	1.97 × 10^−8^	**0**	**0**	**0**
F2	Best	5.84 × 10^−140^	2.16 × 10^−21^	7.95 × 10^−3^	**0**	7.47 × 10^−155^	4.08 × 10^−8^	**0**	**0**	**0**
	Mean	1.20 × 10^−127^	5.58 × 10^−20^	1.69 × 10^−2^	5.78 × 10^−191^	2.73 × 10^−100^	1.17 × 10^−5^	**0**	3.36 × 10^−308^	**0**
	StD	6.45 × 10^−127^	9.78 × 10^−20^	4.79 × 10^−3^	**0**	1.50 × 10^−99^	1.94 × 10^−5^	**0**	**0**	**0**
F3	Best	1.96 × 10^−65^	**0**	5.10 × 10^−16^	**0**	2.3714 × 10^−135^	4.16 × 10^−19^	**0**	**0**	**0**
	Mean	1.01 × 10^−4^	**0**	1.78 × 10^−12^	6.6714 × 10^−129^	8.07 × 10^−9^	5.73 × 10^−11^	**0**	**0**	**0**
	StD	5.53 × 10^−4^	**0**	3.54 × 10^−12^	3.6541 × 10^−128^	1.21 × 10^−9^	1.29 × 10^−10^	**0**	**0**	**0**
F4	Best	1.25 × 10^−92^	4.69 × 10^−15^	0.0245	**0**	5.34 × 10^−156^	2.20 × 10^−8^	**0**	**0**	**0**
	Mean	1.95 × 10^−79^	1.54 × 10^−13^	0.0948	4.11 × 10^−144^	2.23 × 10^−100^	1.44 × 10^−5^	**0**	1.4239E− 301	**0**
	StD	1.06 × 10^−78^	1.47 × 10^−13^	0.0350	2.21 × 10^−143^	1.22 × 10^−100^	2.56 × 10^−5^	**0**	**0**	**0**
F5	Best	**0**	**0**	**0**	**0**	**0**	**0**	**0**	**0**	**0**
	Mean	**0**	**0**	**0**	**0**	**0**	**0**	**0**	**0**	**0**
	StD	**0**	**0**	**0**	**0**	**0**	**0**	**0**	**0**	**0**
F6	Best	**0**	0.7533	0.0001	1.13 × 10^−20^	7.84 × 10^−9^	2.15 × 10^−6^	6.16 × 10^−33^	8.26 × 10^−16^	**0**
	Mean	1.77 × 10^−31^	1.2063	0.0044	3.30 × 10^−15^	2.26 × 10^−5^	3.37 × 10^−4^	4.98 × 10^−24^	2.82 × 10^−14^	**2.67 × 10**^**−33**^
	StD	6.06 × 10^−31^	0.1781	0.0024	1.58 × 10^−14^	5.50 × 10^−5^	6.70 × 10^−4^	2.72864 × 10^−23^	5.74 × 10^−14^	**4.97 × 10**^**−33**^
F7	Best	7.63 × 10^−6^	2.13 × 10^−4^	1.14 × 10^−3^	1.59 × 10^−7^	1.46 × 10^−7^	1.08 × 10^−4^	8.71 × 10^−7^	6.19 × 10^−6^	**2.38 × 10**^**−6**^
	Mean	9.93 × 10^−5^	1.98 × 10^−3^	2.51 × 10^−3^	6.30 × 10^−4^	4.85 × 10^−5^	7.74 × 10^−4^	5.47 × 10^−5^	7.78 × 10^−5^	**4.07 × 10**^**−5**^
	StD	8.49 × 10^−5^	9.81 × 10^−4^	1.25 × 10^−3^	1.11 × 10^−4^	6.61 × 10^−5^	6.13 × 10^−4^	4.92 × 10^−5^	6.77 × 10^−5^	**4.54 × 10**^**−5**^
Multimodal functions										
F8	Best	**− 4189.83**	− 2703.44	− 3594.96	**− 4189.83**	− 4189.67	**− 4189.83**	**− 4189.83**	**− 4189.83**	− 3833.00
	Mean	**− 4189.83**	− 2255.04	− 3059.81	− 4174.04	− 3878.30	− 4097.29	**− 4189.83**	− 4064.56	− 3003.14
	StD	**2.15 × 10**^**−12**^	192.93	262.38	86.50	645.70	351.65	3.73 × 10^−12^	265.98	268.73
F9	Best	− 20.64	− 74.41	− 88.01	**− 90**	**− 90**	**− 90**	**− 90**	**− 90**	**− 90**
	Mean	− 0.69	− 59.95	− 81.66	**− 90**	**− 90**	**− 90**	**− 90**	**− 90**	**− 90**
	StD	3.77	6.63	4.23	**0**	**0**	1.84 × 10–8	**0**	**0**	**0**
F10	Best	− 347.92	− 22003.75	**− 22023.75**	**− 22023.75**	**− 22023.75**	**− 22023.75**	**− 22023.75**	**− 22023.75**	**− 22023.75**
	Mean	− 69.88	− 22003.75	− 22004.44	**− 22023.75**	− 22021.58	− 22007.29	− 22006.02	− 22022.47	− 22013.08
	StD	88.94	**7.40 × 10**^**−12**^	48867365	1.64 × 10^−10^	4.58	7.08	4.73	4.88	10.15
F11	Best	**0**	0.25	0.06	**0**	**0**	**0**	**0**	**0**	**0**
	Mean	**0**	0.60	0.17	6.41E− 05	0.02	**0**	**0**	**0**	**0**
	StD	**0**	0.10	0.06	**0**	3.46E− 12	**0**	**0**	**0**	**0**
F12	Best	**4.71 × 10**^**−32**^	1.57 × 10^−11^	4.27 × 10^−17^	**4.71 × 10**^**−31**^	1.28 × 10^−9^	1.03 × 10^−12^	**4.71 × 10**^**−31**^	**4.71 × 10**^**−31**^	**4.71 × 10**^**−31**^
	Mean	**4.71 × 10**^**−32**^	6.49 × 10^−6^	3.43 × 10^−11^	**4.71 × 10**^**−31**^	1.50 × 10^−6^	2.22 × 10^−7^	**4.71 × 10**^**−31**^	1.07 × 10^−30^	**4.71 × 10**^**−31**^
	StD	**1.67 × 10**^**−47**^	1.33 × 10^−5^	6.92 × 10^−11^	8.91 × 10^−47^	3.11 × 10^−6^	4.48 × 10^−7^	8.91 × 10^−47^	3.29 × 10^−30^	8.91 × 10^−47^
F13	Best	**1.35 × 10**^**−32**^	8.59 × 10^−12^	3.75 × 10^−15^	**1.35 × 10**^**−32**^	1.49 × 10^−11^	1.31 × 10^−11^	**1.35 × 10**^**−32**^	**1.35 × 10**^**−32**^	**1.35 × 10**^**−32**^
	Mean	**1.35 × 10**^**−32**^	5.33 × 10^−6^	6.46 × 10^−12^	**1.35 × 10**^**−32**^	7.04 × 10^−8^	1.42 × 10^−7^	**1.35 × 10**^**−32**^	1.90 × 10^−30^	**1.35 × 10**^**−32**^
	StD	**5.57 × 10**^**−48**^	1.10 × 10^−5^	1.33 × 10^−11^	**5.57 × 10**^**−48**^	1.52 × 10^−7^	2.57 × 10^−7^	**5.57 × 10**^**−48**^	5.76 × 10^−30^	**5.57 × 10**^**−48**^
Fixed− dimensional multimodal function										
F14	Best	**1.00**	**1.00**	**1.00**	**1.00**	**1.00**	**1.00**	**1.00**	**1.00**	**1.00**
	Mean	**1.00**	1.33	**1.00**	1.98	2.43	1.53	**1.00**	3.00	**1.00**
	StD	2.10** × **10^−16^	0.38	1.96E− 13	2.98	3.32	1.26	**4.12 × 10**^**−17**^	3.54	2.90
F15	Best	**3.075 × 10**^**−4**^	7.166 × 10^−4^	3.273 × 10^−4^	3.07493	3.132 × 10^−4^	3.097 × 10^−4^	**3.075 × 10**^**−4**^	3.127 × 10^−4^	**3.075 × 10**^**−4**^
	Mean	**3.075 × 10**^**−1**^	1.163 × 10^−3^	6.392 × 10^−4^	6.32275	4.366 × 10^−4^	3.974 × 10^−4^	3.685 × 10^−4^	6.611 × 10^−4^	3.762 × 10^−3^
	StD	**7.53 × 10**^**−12**^	2.73 × 10^−4^	2.69 × 10^−4^	2.36376	8.266 × 10^−5^	7.429 × 10^−5^	2.323 × 10^−4^	4.906 × 10^−4^	7.558 × 10^−2^
F16	Best	**− 1.032**	**− 1.032**	**− 1.032**	**− 1.032**	**− 1.032**	**− 1.032**	**− 1.032**	**− 1.032**	**− 1.032**
	Mean	**− 1.032**	**− 1.032**	**− 1.032**	**− 1.032**	**− 1.032**	**− 1.032**	**− 1.032**	**− 1.032**	**− 1.032**
	StD	**6.25 × 10**^**−16**^	3.54 × 10^−5^	3.29 × 10^−9^	6.71 × 10^−16^	1.57 × 10^−4^	3.33 × 10^−6^	6.71 × 10^−16^	6.90 × 10^−3^	6.65 × 10^−16^
F17	Best	**0.398**	**0.398**	**0.398**	**0.398**	**0.398**	**0.398**	**0.398**	**0.398**	**0.398**
	Mean	**0.398**	0.542	**0.398**	**0.398**	**0.398**	**0.398**	**0.398**	**0.398**	**0.398**
	StD	**0**	0.725	2.16 × 10^−8^	**0**	1.55 × 10^−4^	9.02 × 10^−9^	**0**	1.62 × 10^−9^	**0**
F18	Best	**3**	> 3	**3**	**3**	> 3	> 3	**3**	> 3	**3**
	Mean	**3**	> 3	> 3	**3**	> 3	> 3	**3**	12.9850033	3.9
	StD	1.9710^−15^	4.26** × **10^−4^	1.22** × **10^−8^	3.92** × **10^−15^	2.27** × **10^−2^	2.144** × **10^−5^	**8.96 × 10**^**−16**^	21.94	4.93
F19	Best	**− 3.863**	− 3.854	**− 3.863**	**− 3.863**	**− 3.863**	**− 3.863**	**− 3.863**	**− 3.863**	**− 3.863**
	Mean	**− 3.863**	− 3.787	**− 3.863**	**− 3.863**	− 3.856	− 3.818	**− 3.863**	− 3.825	**− 3.863**
	StD	2.58 × 10^−15^	0.263	3.20 × 10^−9^	2.71 × 10^−15^	2.84 × 10^−3^	6.94 × 10^−2^	**2.682 × 10**^**−15**^	0.142	2.696 × 10^−15^
F20	Best	**− 3.322**	− 3.009	**− 3.322**	**− 3.322**	− 3.295	− 3.187	**− 3.322**	**− 3.322**	**− 3.322**
	Mean	− 3.267	− 2.751	− 3.299	− 3.282	− 3.208	− 2.831	**− 3.290**	− 2.888	− 3.263
	StD	0.06	0.39	**0.05**	0.07	0.08	0.33	**0.05**	0.62	0.06
F21	Best	**− 10.153**	− 5.69	**− 10.153**	**− 10.153**	**− 10.153**	− 5.055	**− 10.153**	**− 10.153**	**− 10.153**
	Mean	**− 10.153**	− 3.221	− 9.157	− 9.983	− 10.149	− 5.045	**− 10.153**	**− 10.153**	− 7.184
	StD	6.02 × 10^−15^	1.278	2.58	0.93	0.01	0.02	6.68 × 10^−15^	5.55 × 10^−4^	2.68
F22	Best	**− 10.403**	− 6.306	**− 10.403**	**− 10.403**	**− 10.403**	− 5.088	**− 10.403**	**− 10.403**	**− 10.403**
	Mean	**− 10.403**	− 2.917	− 9.541	**− 10.403**	− 10.399	− 5.068	**− 10.403**	− 10.224	− 6.073
	StD	**6.60 × 10**^**−16**^	1.20	2.28	7.34 × 10^−15^	0.01	0.05	9.33 × 10^−16^	0.97	2.790
F23	Best	**− 10.536**	− 4.055	**− 10.536**	**− 10.536**	**− 10.536**	− 5.129	**− 10.536**	**− 10.536**	**− 10.536**
	Mean	**− 10.536**	− 2.838	**− 10.536**	**− 10.356**	− 10.533	− 5.119	**− 10.536**	**− 10.236**	− 6.210
	StD	1.32 × 10^−15^	0.61	7.71 × 10^−6^	0.99	0.01	0.01	**9.33 × 10**^**−16**^	1.166	2.81
Results	Best	15	5	10	19	11	9	21	19	**22**
	Mean	14	3	6	13	4	5	**20**	9	16
	StD	10	3	2	7	3	2	**14**	7	10

The best results are marked in bold.

**Figure 8:**
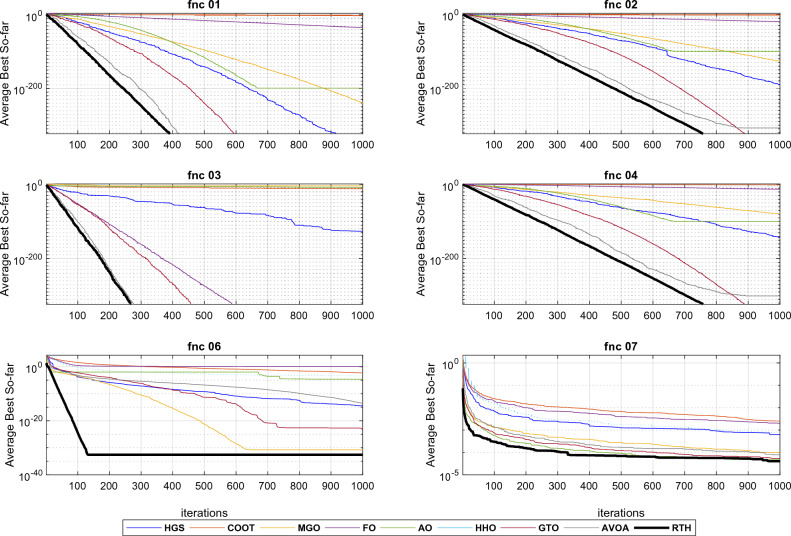
Convergence curves of the unimodal functions.
- *Multimodal functions results (F 08–13)* Multimodal test functions include multiple local solutions. Their number grows exponentially as the number of search space dimensions grows (optimization variables). Having more than one optimal is beneficial if the purpose is to test an algorithm's exploration capabilities. As a result, they are well-suited for assessing optimization algorithms' local optima avoidance and exploratory behavior. The obtained results in Table 3 and Fig. 9 demonstrated that RTH has superior exploration capabilities to other methods for five of six functions in terms of finding the optimal solution with the lowest number of iterations. These functions approve the ability of the RTH to handle complicated problems.

- *Unimodal functions results (F 01–07)* because unimodal test functions have just one global optimum solution, they may be used to assess an algorithm's convergence speed (exploitation phase). From Table 3 results and the curves illustrated in Fig. 8 (fnc 05 curves are not included due to the exact similarity in the results), the RTH outperformed all algorithms in all test functions in terms of accuracy and convergence speed. These results demonstrate RTH's capabilities in quick exploitation, which can enable RTH in fast convergence. This capacity is derived from the high soaring phase that uses the adaptive transition factor (TF) and Lévy distribution.

**Figure 9:**
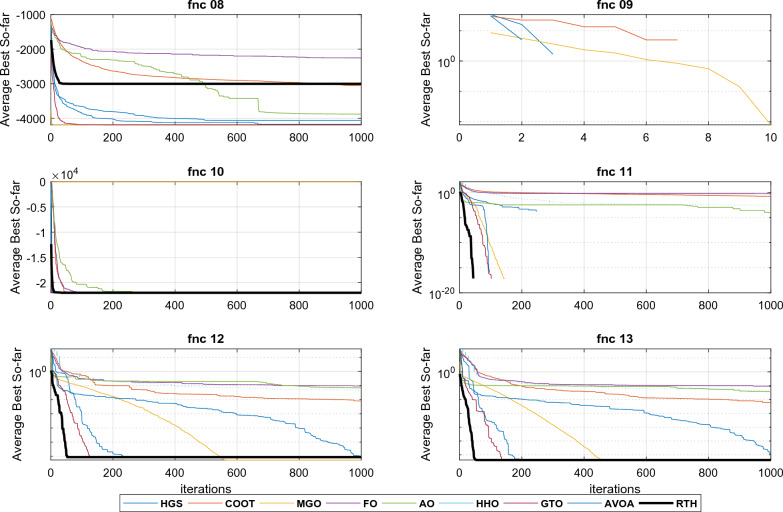
Convergence curves of the multimodal functions.
- *Fixed-dimensional multimodal function (F 14–23)* similar to the multimodal test functions, these functions include a lot of local optima with more complexity due to the minimization of the negative cost function. Hence, they are suitable for evaluating the accuracy of the algorithm. As illustrated in Fig. 10 and reported in Table 3, the proposed RTH has reached the global optimum for all the considered functions. However, its robustness, represented by the standard deviation (StD), has been reduced to the increased complexity. RTH's exploration is due to its different optimization phases and the acceleration and gravity effects.

**Figure 10:**
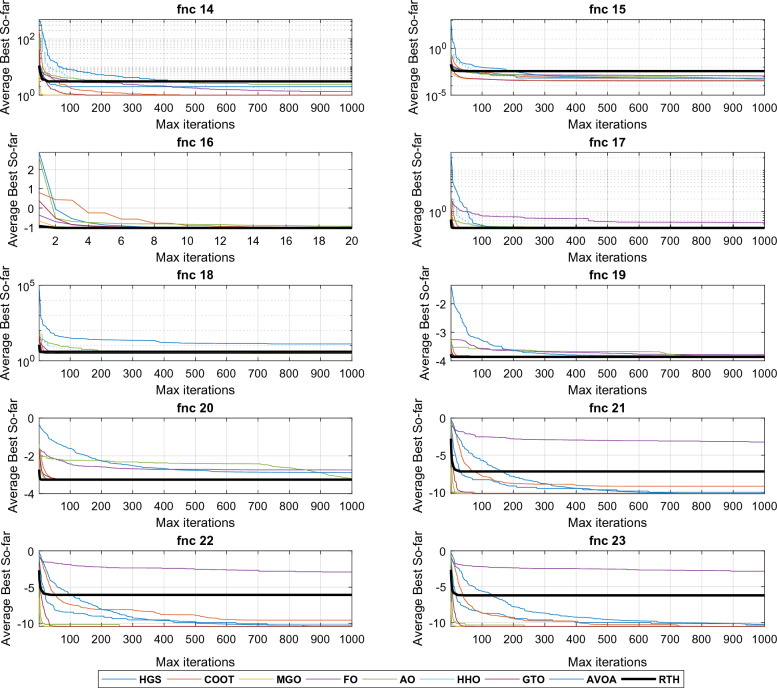
Convergence curves of the multimodal functions.

### IEEE CEC 2020

**Figure 11:**
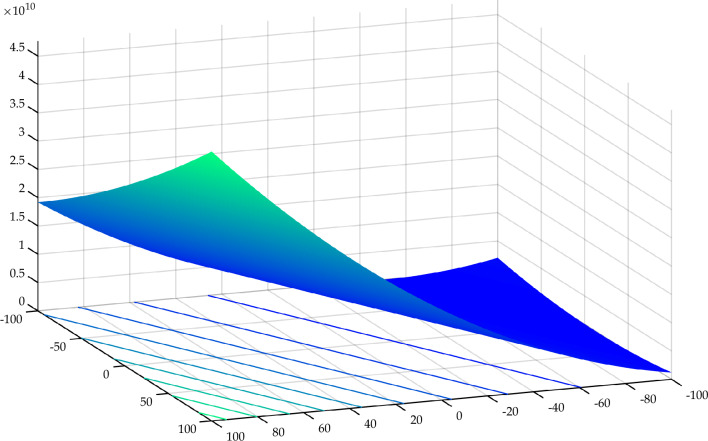
CEC 2020 F1 (unimodal) 3D map.

**Figure 12:**
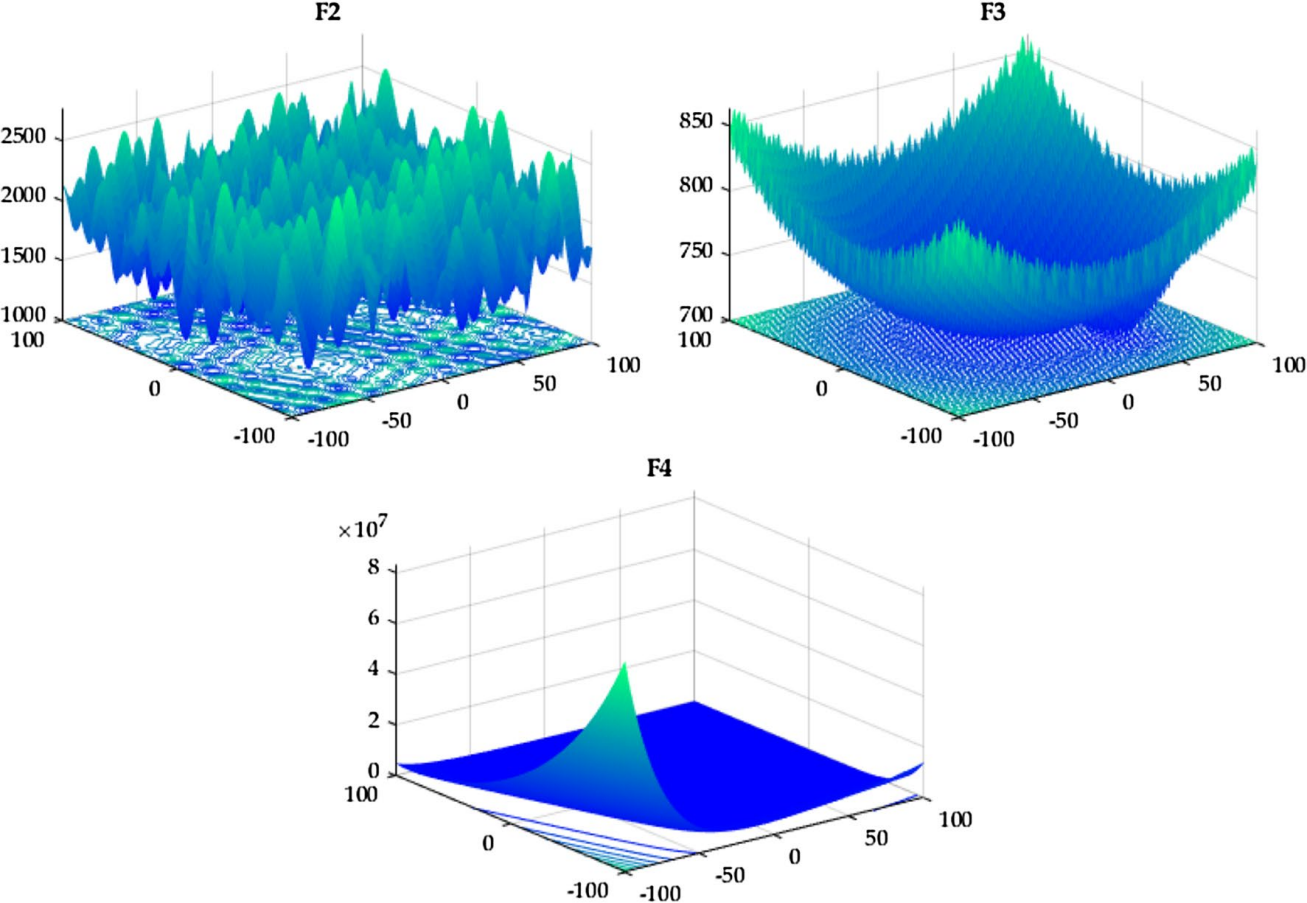
CEC 2020 basic functions’ 3D map (F2–F4).

**Figure 13:**
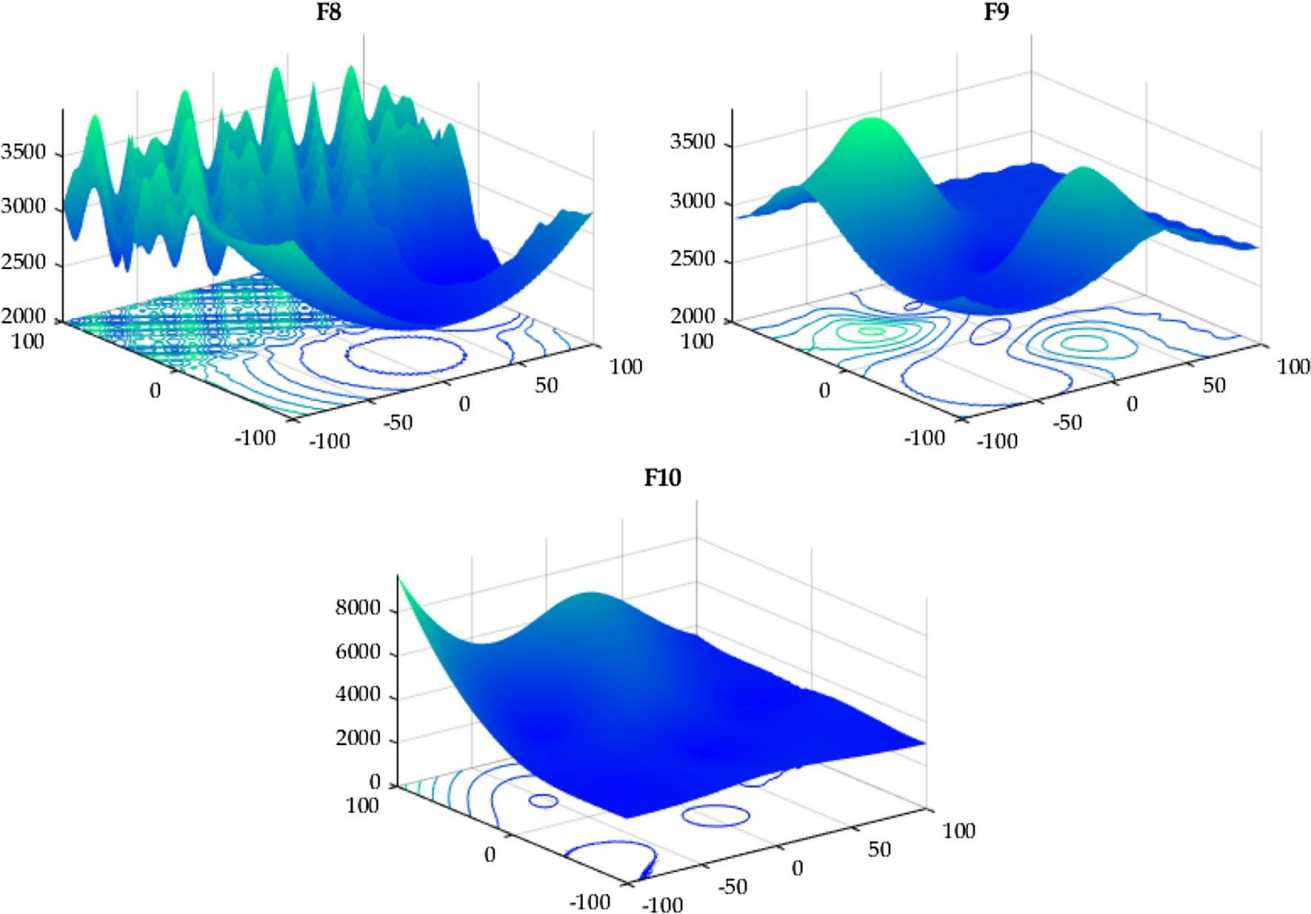
CEC 2020 composition functions’ 3D map (F8–F10).

The IEEE Congress on Evolutionary Computation (CEC) offers yearly benchmark functions to test and evaluate optimization algorithms' ability to identify optimal solutions (minimization problems). This collection of functions is commonly recognized by the year as standard functions established by the IEEE Congress on Evolutionary Computation, for example, CEC 2020 and CEC 2022. These test functions include unimodal, basic multimodal, hybrid, and composition functions. First, the proposed RTH will be compared with the considered algorithms using the ten functions of the IEEE CEC 2020^55^. The characteristics of CEC 2020 functions are presented in Table A2. Their 3D maps are shown in Fig. 11 for the unimodal function (F1), Fig. 12 for the basic functions (F2-F4), and Fig. 13 for the composition functions (F8–F10).

**Figure 14:**
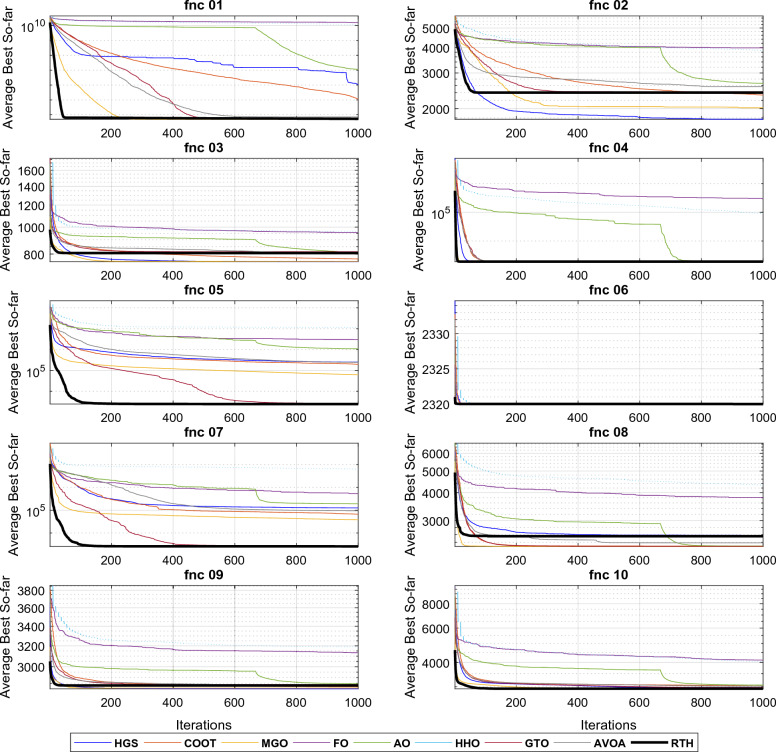
CEC 2020 fitness evolution for D = 15.

All the algorithms are run 30 times for each function. The results for these functions for D = 15 are presented in Table 4 and. These include the best, the main, and the standard deviation (StD). The fitness evolutions during the optimization process for each function are presented in Fig. 14.

**Table 4:** CEC 2020 benchmark test function results with D = 15.

		MGO	FO	COOT	HGS	AO	HHO	GTO	AVOA	RTH
Unimodal function										
F1	Best	154.539	8576.36 × 10^6^	41887	2181	4024949	4911.13 × 10^6^	104.345	102.058	**100.592**
	Mean	4974.367	1610.29 × 10^7^	112335	1135972	11527453	1208.31 × 10^7^	5013.844	7186.27	**4365.139**
	StD × 10^3^	4.779	2.867 × 10^6^	45.662	4.50 × 10^3^	8.05 × 10^3^	3.55 × 10^6^	5.93	8.32	**4.00**
Basic functions										
F2	Best	**1126.91**	3620.76	1572.07	1412.69	2007.40	2996.31	1678.96	1925.81	1571.65
	Mean	**2020.35**	3987.02	2334.50	1773.44	2667.60	3857.45	2423.14	2569.96	2401.16
	StD	382.45	**184.61**	260.14	245.91	387.24	363.03	404.35	294.22	430.07
F3	Best	**723.00**	911.14	741.18	725.33	774.33	895.80	746.42	766.98	751.74
	Mean	**752.73**	955.17	768.25	751.06	809.70	963.07	815.69	810.54	806.92
	StD	19.811	19.78	**14.66**	17.71	21.97	31.88	34.12	32.44	33.36
F4	Best	1901.38	70470.76	1902.476	1901.19	1909.34	1958.76	1902.26	1902.13	**1900.08**
	Mean	1903.72	294336.46	1906.02	1908.62	1918.19	91339.86	1908.18	1908.71	**1903.69**
	StD	**1**	200755.19	1.98	7.30	9.88	82844.70	4.70	3.65	2.66
Hybrid functions										
F5	Best	2492.57	755289	14050	13508.5	96570.2	1270090	1986.35	3909.53	**2015.57**
	Mean	62543.12	2969214	196629	249879	1098805	11324500	2563.61	244561	**2439.74**
	StD	57658	1323317	139143	325361	1047277	12488412	286.09	169180	**249.82**
F6	Best	**2319.99**	**2319.99**	**2319.99**	**2319.99**	**2319.99**	**2319.99**	**2319.99**	**2319.99**	**2319.99**
	Mean	**2319.99**	**2319.99**	**2319.99**	**2319.99**	**2319.99**	**2319.99**	**2319.99**	**2319.99**	**2319.99**
	StD × 10^−3^	4.47 × 10^−8^	3.96 × 10^−2^	8.27 × 10^−6^	**1.39 × 10**^**−9**^	6.48 × 10^−3^	0.115	**1.39 × 10**^**−9**^	**1.39 × 10**^**−9**^	**1.39 × 10**^**−9**^
F7	Best	2269.802	164541.7	4580.516	5310.281	7155.679	852667.0	**2136.214**	3331.492	2161.322
	Mean	38399.91	527133.1	66495.89	125533.1	191838.5	6232589	2581.630	94355.00	2597.401
	StD	41656.35	241929.1	88216.10	119168.3	151067.9	3376108	208.5411	108669.8	239.2218
Composition functions										
F8	Best	2300	3107.01	2302.31	2300.51	2308.68	2980.88	2300	**2299.52**	2300
	Mean	**2300.96**	3811.25	2304.30	2578.74	2311.52	4326.10	2304.54	2388.05	2556.90
	StD	**0.653**	381.30	1.44	445.75	0.81	963.94	6.87	338.93	678.38
F9	Best	2796.06	3059.19	2501.81	**2500**	2550.93	2858.15	2805.19	2801.05	2800
	Mean	2808.72	3133.14	2819.69	**2801.70**	2846.57	3191.31	2839.22	2836.49	2831.28
	StD	**7.79**	41.27	62.36	57.56	59.29	138.81	46.35	21.04	15.06
F10	Best	2900	3628.14	2901.42	2900	2909.24	3482.782	2900	2900	**2900**
	Mean	2935.55	4107.85	3019.66	2973.60	3059.41	4035.529	2975.479	3021.413	**2920.14**
	StD	84.64	316.40	97.67	100.56	84.6	309.486	102.096	102.84	**61.44**
L1	Best	3	1	1	2	1	1	2	2	**5**
L2	Mean	4	1	1	2	1	1	1	1	**5**
L3	StD	3	1	1	1	0	0	1	1	**4**

The best results are marked in bold.

The three last rows show the scores of each algorithm. It can be seen from the L1 row that the proposed RTH can find the optimal solution for five functions from ten, followed by MGO by three functions, then HGS, AVOA, and GTO by two functions, where the other algorithms achieved only one optimal solution. From the penultimate row, the proposed RTG can accomplish the best mean results for five problems, followed by MGO by four, then HGS by two. The last row presents the StD results that confirm the algorithm's robustness. Based on these results, the proposed RTH provided the best results in this criterion four times. The evolution of the average cost function is presented in Fig. 13. From this figure, the proposed RTH has fast convergence speed compared to the other algorithms. This can be explained by the unique exploitation and exploitation strategies of the proposed RTH algorithm.

The functions of the CEC 2020 benchmark will be tested with increased search space dimension where D is equal to 20. This allows us to investigate the performance with more complex cases. The results are provided in Table 5.

**Table 5:** CEC 2020 benchmark test function results with D = 15.

		MGO	FO	COOT	HGS	AO	HHO	GTO	AVOA	RTH
Unimodal function										
F1	Best	103.05	16806140527	224340.05	130.33	7001885.6	13513137381	100.25	100.49	**100.20**
	Mean	2840.68	29506906789	637140.05	10976.98	39584617.2	21692845681	2554.76	3266.99	**1715.68**
	StD × 10^3^	3312.12	4605945120	311729.08	16272.32	21104674.4	4650504699	2610.12	3863.87	**1565.21**
Basic functions										
F2	Best	1472.78	4746.85	2112.23	**1455.18**	2653.59	4630.30	2276.34	1926.65	1966.20
	Mean	2333.59	5471.61	3206.10	**1905.89**	3385.10	5383.45	3238.12	2838.21	3156.77
	StD	438.85	259.64	452.76	**220.67**	474.10	357.14	463.24	482.15	596.00
F3	Best	743.17	982.24	776.23	**735.22**	803.50	931.26	798.78	785.60	796.21
	Mean	783.98	1061.86	815.46	**762.18**	855.62	1028.41	859.93	836.36	844.61
	StD	20.88	36.71	28.20	**17.65**	27.78	31.34	41.12	30.18	30.29
F4	Best	1902.61	98274.78	1905.98	1901.75	1915.55	9210.16	1905.96	1906.25	**1900.93**
	Mean	1910	358004.03	1910.40	1903.68	1932.14	227855.65	1918.30	1914.45	**1909.82**
	StD	9.30	246927.96	2.57	1.20	12.14	190896.04	9.38	6.26	**4.61**
Hybrid functions										
F5	Best	13793.98	963554.04	71241.37	35642.41	180500.1	258875.09	4762.99	25666.73	**2793.50**
	Mean	103507.19	3221412.17	384815.44	769776.7	640537.1	3585480.15	32023.92	480253.8	**6392.68**
	StD	60422.30	1429844.08	239613.83	527020.1	339804.200	2798617.66	34478.79	413365.6	**3120.73**
F6	Best	**1604.05**	**1604.05**	**1604.05**	**1604.05**	**1604.05**	**1604.05**	**1604.05**	**1604.05**	**1604.05**
	Mean	**1604.05**	**1604.05**	**1604.05**	**1604.05**	**1604.05**	**1604.05**	**1604.05**	**1604.05**	**1604.05**
	StD × 10^−12^	**1.16**	**1.16**	**1.16**	**1.16**	**1.16**	**1.16**	**1.16**	**1.16**	**1.16**
F7	Best	3544.55	271391.75	9867.50	34895.32	42748.08	318315.91	2621.89	19735.31	**2443.60**
	Mean	65648.76	763153.83	144945.05	284910.30	530727.72	3573597.72	4921.82	226243.15	**3648.84**
	StD	57276.90	423466.26	92024.16	253278.10	450075.13	3473899.96	1940.52	189687.04	**947.33**
Composition functions										
F8	Best	**2300**	4347.34	2306.97	**2300**	2314.95	3880.60	**2300**	**2300**	**2300**
	Mean	**2300.70**	5396.11	2309.54	3209.51	2321.03	6004.87	2418.29	2768.84	2671.51
	StD	**0.63**	576.35	1.13	1247.79	4.81	892.123	639.17	1107.05	1004.51
F9	Best	**2821.03**	3125.32	2821.07	2850.66	2863.87	3081.44	2837.50	2860.88	2861.11
	Mean	**2853.78**	3281.45	2883.40	2917.45	2927.31	3341.05	2905.20	2948.24	2918.42
	StD	**26.25**	59.29	37.24	34.10	44.59	149.48	49.74	49.63	40.15
F10	Best	2913.93	4160.30	2911.42	**2909.25**	2941.17	3605.15	2913.10	2910.51	2901.27
	Mean	2973.47	4890.83	2979.09	**2931.18**	3010.99	4473.18	2969.76	2982.71	2959.72
	StD	33.02	539.25	31.04	**28.29**	23.33	643.61	35.28	34.40	31.12
L1	Best	3	1	1	2	1	1	2	2	**6**
L2	Mean	3	1	1	2	1	1	1	1	**5**
L3	StD	2	1	1	2	1	1	1	1	**5**

The best results are marked in bold.

**Figure 15:**
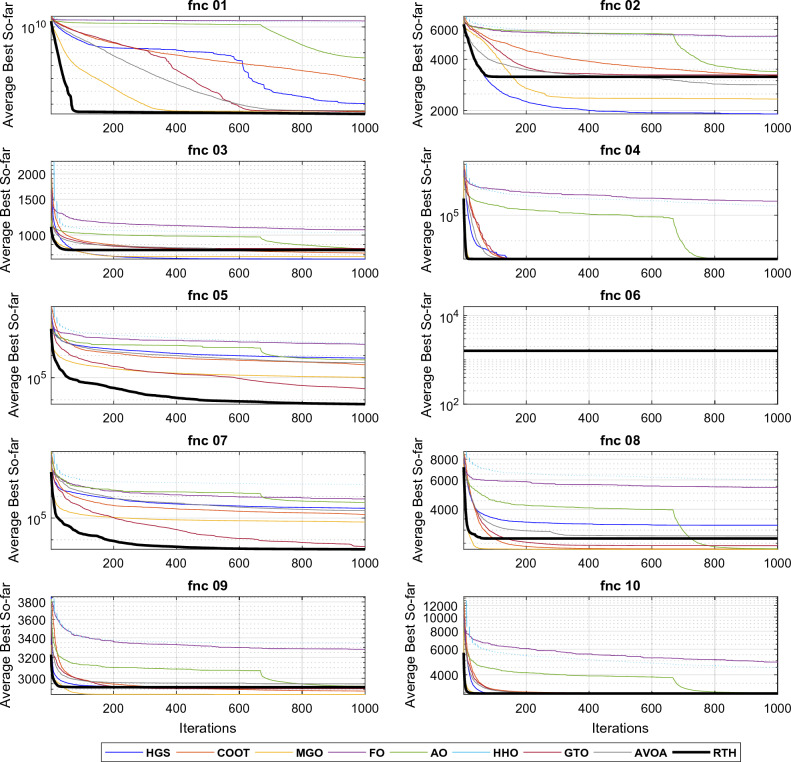
CEC 2020 fitness evolution for D = 20.

It can be seen from the L1 row that the proposed RTH can find the optimal solution for six functions, followed by MGO by three, then HGS, AVOA, and GTO by two functions. From the penultimate row, the proposed RTG has got the best mean results for five problems, followed by MGO by three, then HGS by two. From the last row, the StD results of the RTH is the bet by five times, which confirms the algorithm's robustness. Based on these results, the proposed RTH provided the best results in this criterion four times. The evolution of the average cost function is presented in Fig. 15. Similar to the previous case, the proposed RTH has fast convergence speed compared to the other algorithms.

ANOVA is the abbreviation for analysis of variance. ANOVA is frequently used to evaluate equality across different means by comparing variance between groups with variation within groups. This test will be used to assess the results between the optimizers for both D = 15 and D = 20 cases. The best outcome for each case is marked in red.

**Figure 16:**
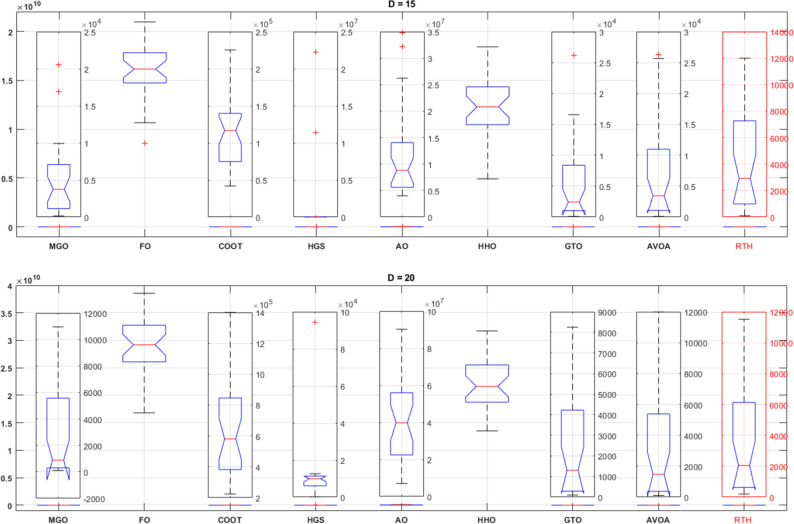
CEC 2020 unimodal function (F1) ANOVA results.

For the unimodal function (function 01), the ANOVA ranking is presented in Fig. 16. From this figure; the RTH generates the best solutions in terms of accuracy with the lowest variation range. On the other hand, the FO and the HHO cannot find any solution.

**Figure 17:**
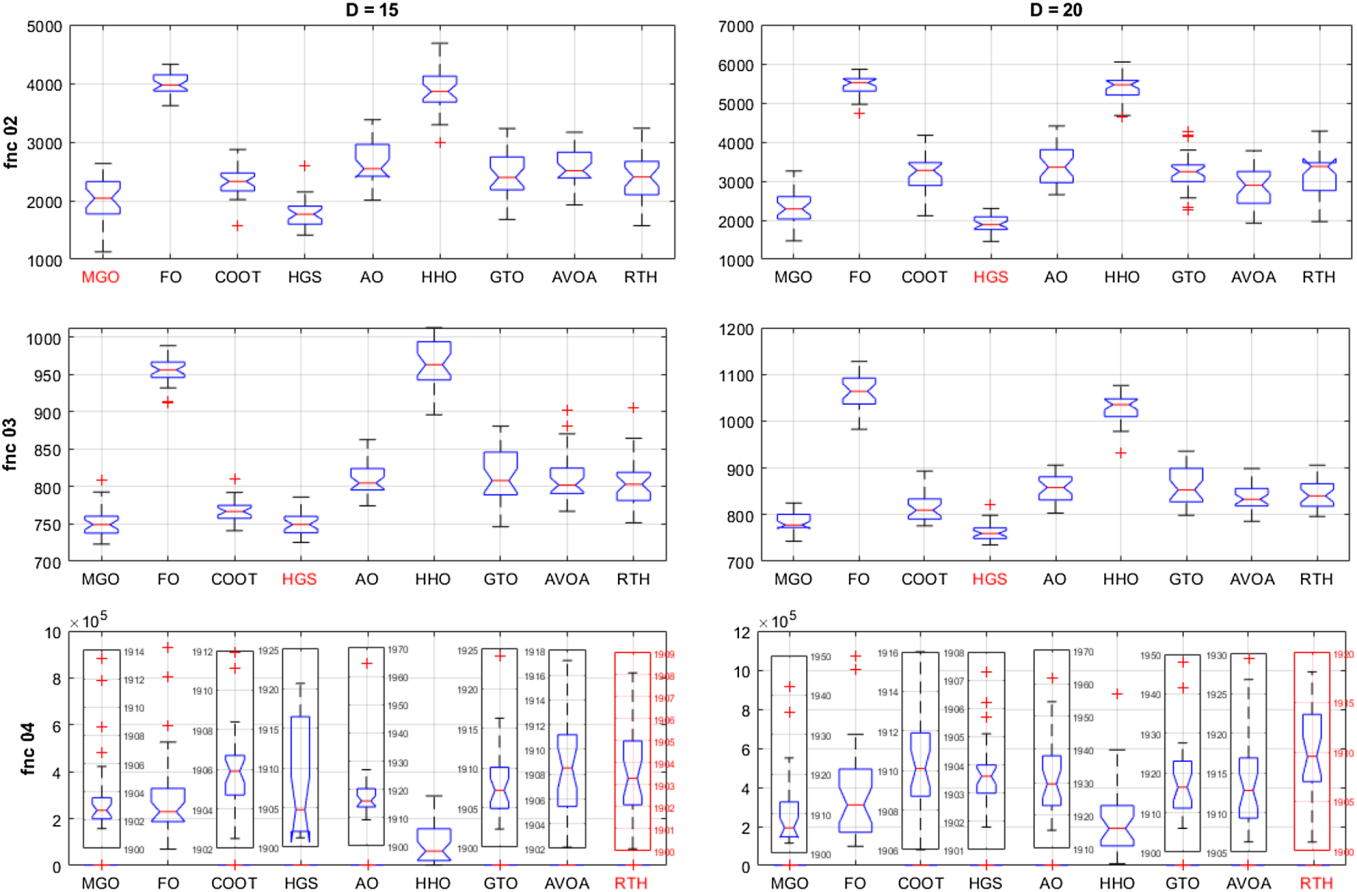
CEC 2020 basic functions ANOVA results.

For the basic functions (fnc 02–04), their results are presented in Fig. 17. For fnc 02 for both cases (D = 15 and D = 20), all the optimizers except for the F and the HHO got the optimal solution where the COOT provided the lower variation range and the MGO include the set of the best result. The results of function 03 are similar to those of function 02, with slight superiority to the HGS in precision and stability. For function 04, the RTH provided identical results for both cases (D = 15 and D = 20). This confirms its ability to resolve this problem effectively.

**Figure 18:**
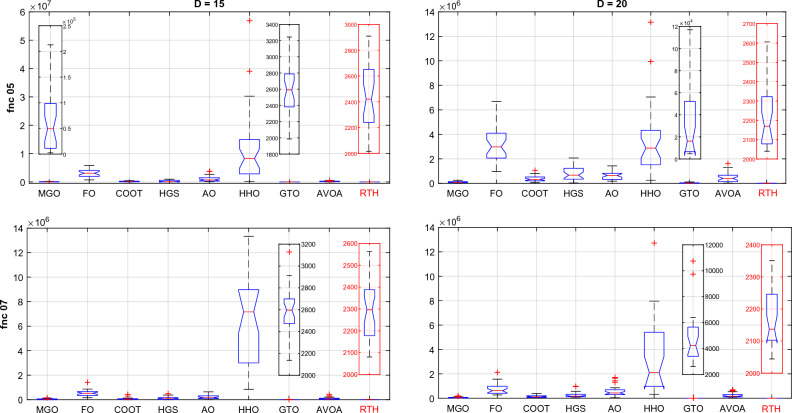
CEC 2020 hybrid functions ANOVA results.

Hybrid functions (fnc 05–07) ANOVA results are presented in Fig. 18. The results of function 06 are not included because of the exact similarity of the results. These figures confirm the outperformance of the proposed RTH for the three functions compared to the other considered algorithms. The MGO and GTO algorithms performed the second best after the RTH.

**Figure 19:**
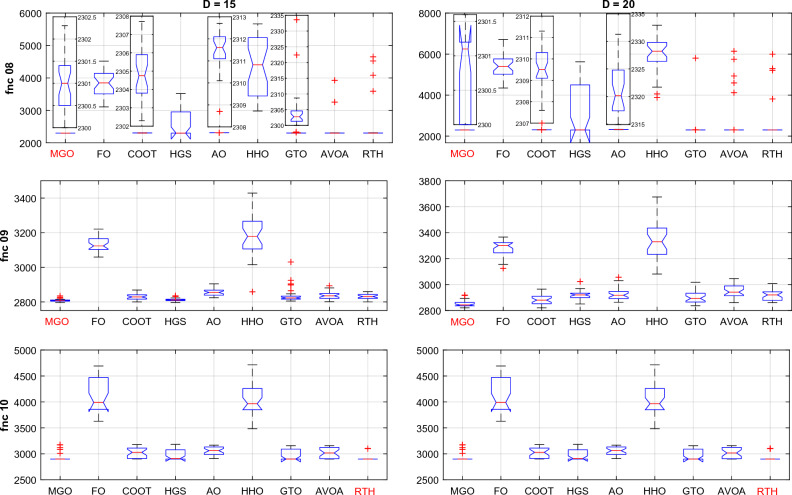
CEC 2020 composite functions ANOVA results.

Composition functions (fnc 08–10) ANOVA results are presented in Fig. 19. For function 08; the MGO provided the best performance where the proposed RTH for the best result, but its variation range is higher due to some ultimate results far from the best. The performance of the algorithms is much closer to each other except for the FO and the HHO for function 09, with light superiority for the MGO. The RTH provides more stable performance and higher accuracy for function 10, followed by the MGO.

#### IEEE CEC 2022

**Figure 20:**
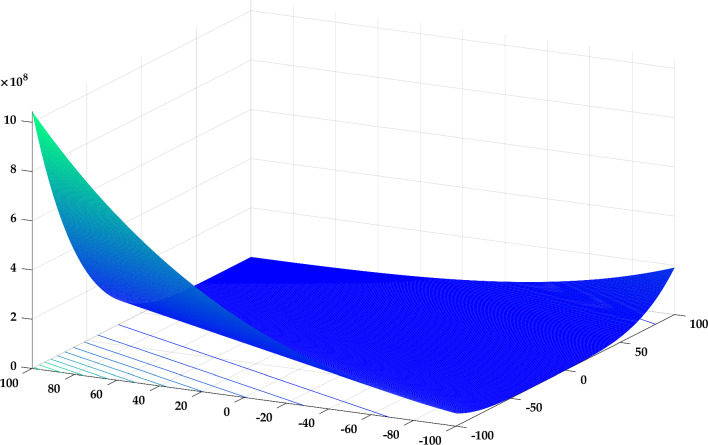
CEC 2022 F1 (unimodal) 3D map.

**Figure 21:**
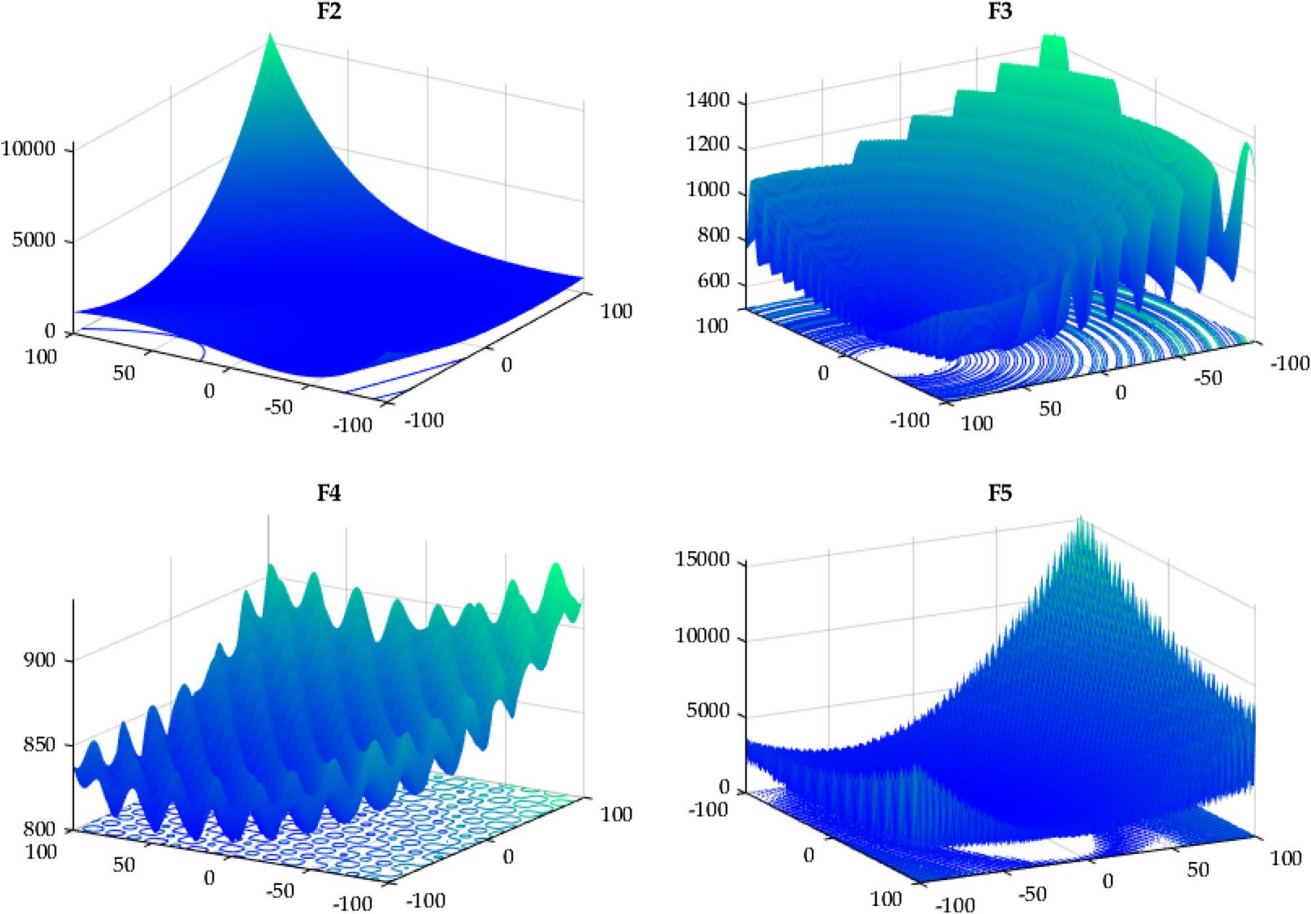
CEC 2022 basic functions’ 3D map (F2–F5).

**Figure 22:**
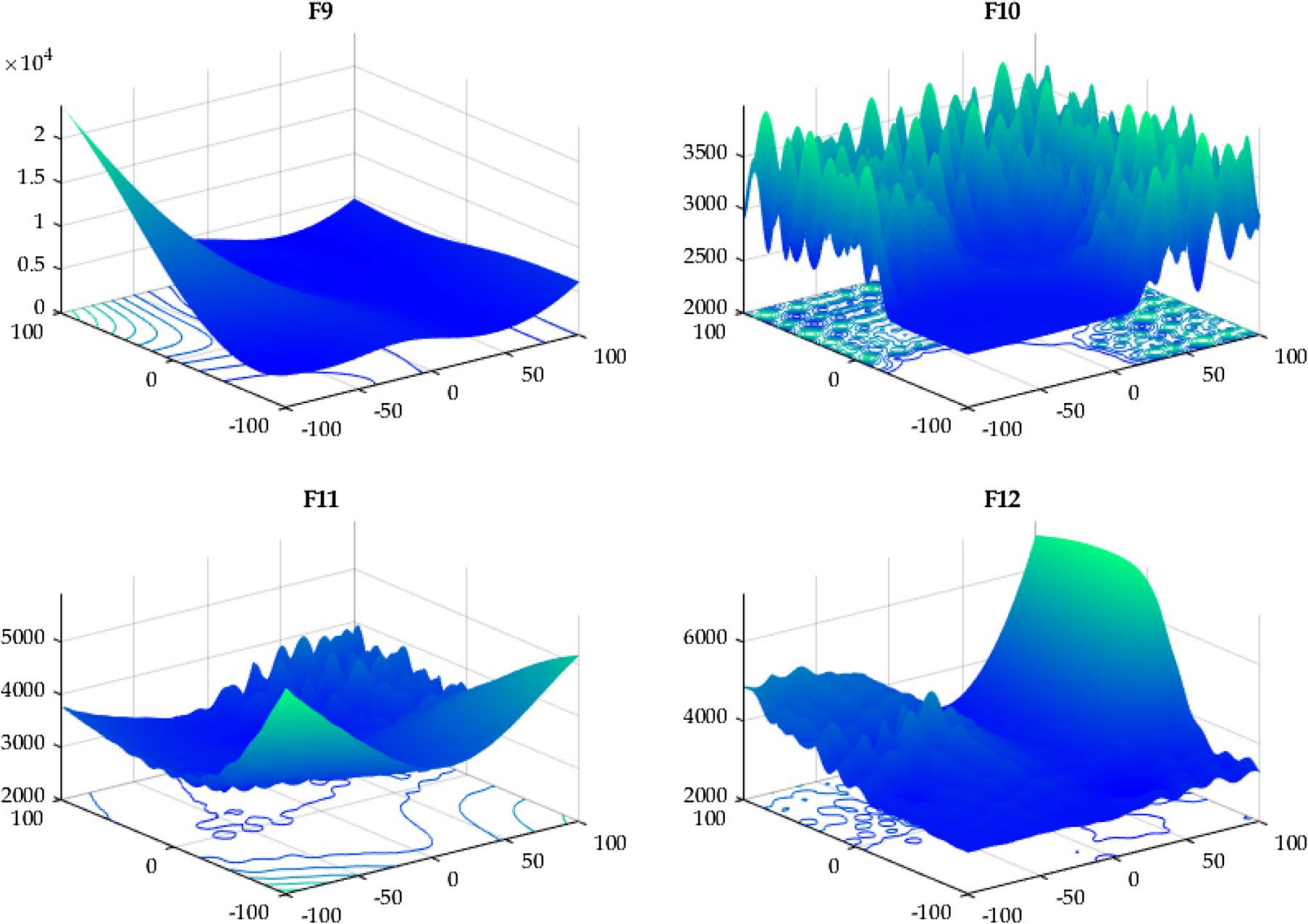
CEC 2022 composition functions’ 3D map (F9–F12).

In this case, the proposed RTH will be compared with the considered algorithms using the 12 functions of the IEEE CEC 2022^56^. The characteristics of CEC 2022 functions are presented in Table A3. Their 3D maps are illustrated in Fig. 20 for the unimodal function (F1), Fig. 21 for the basic functions (F2-F5), and Fig. 22 for the composition functions (F9-F12).

Similar to the CEC 2020 cases, all the algorithms are run 30 times for each function for D = 10 and D = 20. The obtained results for these functions for D = 21 are presented in Table 6.

**Table 6:** CEC 2022 benchmark test function results for D = 10.

		MGO	FO	COOT	HGS	AO	HHO	GTO	AVOA	RTH
Unimodal function										
F1	Best	**300**	7075.29	300.23	**300**	439.34	5774.01	**300**	**300**	**300**
	Mean	**300**	12242.75	302.53	321.05	2127.41	9833.45	**300**	302.72	**300**
	StD	4.09 × 10^−12^	3256.05	2.71	115.26	1730.22	1056.31	1.93E-10	8.05	**3.95 × 10**^**−14**^
Basic functions										
F2	Best	400.001	519.53	400.002	400.39	400.189	502.241	400.002	400.013	**400**
	Mean	405.32	765.59	419.209	421.58	421.727	850.32	408.968	415.57	**404.022**
	StD	3.77	153.57	28.72	29.89	36.835	321.15	17.82	25.613	**3.25**
F3	Best	**600**	633.70	600.40	**600**	605.12	626.52	601.18	602.62	600.82
	Mean	**600.17**	643.93	603.26	600.67	618.32	648.29	607.01	617.85	611.97
	StD	**0.25**	5.38	2.44	0.60	6.92	11.60	5.17	10.68	9.77
F4	Best	**802.99**	839.46	807.34	813.93	810.07	831.72	808.96	808.96	810.95
	Mean	**813.73**	856.94	818.33	838.07	823.00	844.06	827.20	833.13	823.68
	StD	7.25	6.92	**5.92**	14.68	8.21	9.92	7.41	11.01	8.90
F5	Best	**900**	1170.61	900.01	900.91	903.56	1142.71	902.40	955.37	902.36
	Mean	**904.88**	1375.01	902.57	1141.03	993.59	1537.75	956.74	1186.98	1053.05
	StD	**9.07**	124.92	4.16	252.20	81.42	205.56	66.27	196.57	174.49
Hybrid functions										
F6	Best	1846.46	430643.97	1917.56	1835.65	3197.23	3007.22	1805.15	1877.29	**1802.74**
	Mean	2122.37	8578353.07	3444.352	6035.25	10648.95	2588374.51	2008.43	3592.39	**1840.81**
	StD	344.20	7161565.78	1736.32	2355.11	5945.94	8077679.39	707.50	1969.34	**57.95**
F7	Best	2001	2066.31	2009.90	**2000.01**	2021.64	2039.65	2006.52	2020.59	2012.93
	Mean	2020.96	2094	2029.99	**2016.77**	2048.19	2106.54	2029.45	2038.85	2038.61
	StD	**6.29**	11.28	9.91	7.44	24.12	30.63	11.62	20.07	25.97
F8	Best	2214.70	2233.23	2208.56	2219.43	2208.16	2228.73	2206.81	2221.88	**2211.59**
	Mean	2226.69	2246.53	2224.47	2222.47	2227.78	2249.43	2221.81	2225.57	**2221.34**
	StD	21.87	6.53	4.02	3.47	6.11	24.51	3.39	3.35	**2.31**
Composition functions										
F9	Best	**2529.28**	2635.33	**2529.28**	**2529.28**	2530.96	2634.54	**2529.28**	**2529.28**	**2529.28**
	Mean	**2529.28**	2680.84	2529.29	2531.32	2587.69	2730.09	2529.45	2534.18	**2529.28**
	StD	**0**	21.64	0.03	11.14	32.55	45.59	0.59	26.83	**0**
F10	Best	2500.22	2509.88	2500.33	2500.41	2500.71	2502.28	2500.31	2500.37	**2500.10**
	Mean	**2531.19**	2528.46	2546.63	2557.65	2588.60	2812.47	2527.34	2564.39	2547.56
	StD	52.07	**10.33**	57.59	61.64	54.02	455.78	49.60	65.20	62.76
F11	Best	**2600**	2822.82	2600.39	**2600**	2604.60	2787.33	**2600**	**2600**	**2600**
	Mean	2721.97	2946.83	2731.62	2805.30	2690.65	3284.55	**2638.41**	2719.58	2739.04
	StD	123.03	**81.79**	183.31	170.69	93.38	406.47	88.85	138.41	147.85
F12	Best	2861.41	2884.28	2859.00	2861.41	2862.75	2873.20	2861.44	2861.41	**2860.18**
	Mean	2864.73	2905.10	2864.85	**2864.07**	2867.99	2985.81	2864.64	2866.86	2866.71
	StD	1.49	13.50	2.33	**1.43**	3.45	78.94	2.13	5.65	5.74
L1		6	0	1	5	0	0	3	4	**8**
L2		**6**	0	0	2	0	0	2	0	5
L3		4	2	0	1	0	0	0	0	**5**

The best results are marked in bold.

**Figure 23:**
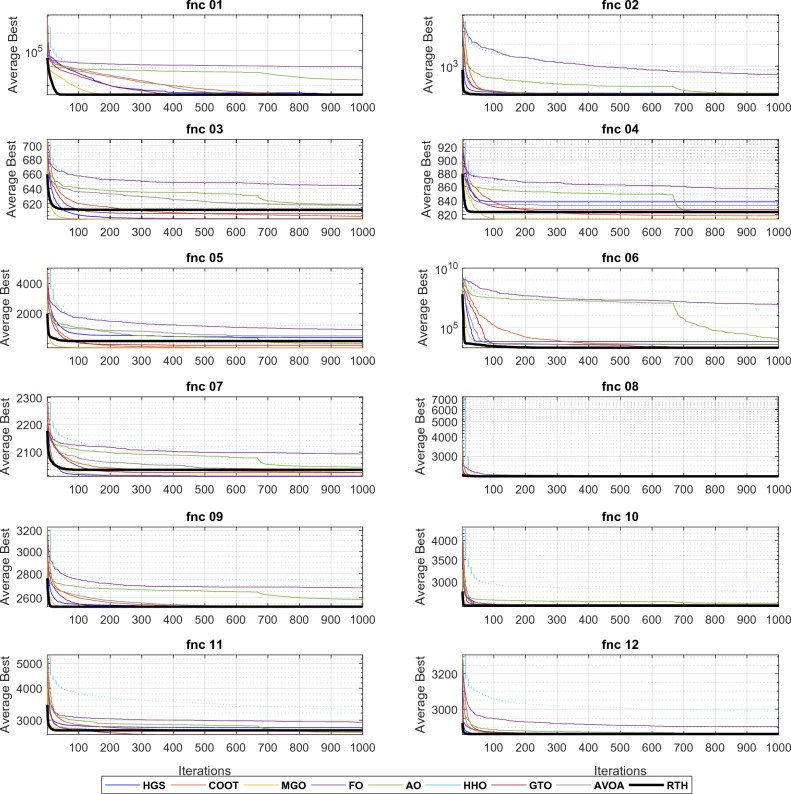
CEC 2022 fitness evolution for D = 10.

From the L1 row, the proposed RTH can find the optimal solution for eight from twelve functions, followed by GTO by six functions, then the HGS by five functions, then AVOA by four. This can approve its ability to get the optimal solution and escape from the local solutions. In comparison, FO, AO, and HHO cannot provide any best solution. From the penultimate row, the MGO achieved the best mean results by six functions, followed by the proposed RTH by five functions, then the HGS and GTO by two. This approves the accuracy of the proposed RTH. FO, COOT, AO, HHO, and AVOA algorithms didn't get any mean best results for all the tests. From the last row, the proposed RTH provided the best results by five functions, followed by the proposed MGO by four times. This can approve its robustness. Figure 23 presents the fitness evolution for each function of the CEC 2022 with D = 10. These figures support the findings of the CEC 2020, where the proposed RTH gives an excellent convergence speed.

For D = 20 case, the results are provided in Table 7.

**Table 7:** CEC 2022 benchmark test function results for D = 10.

		MGO	FO	COOT	HGS	AO	HHO	GTO	AVOA	RTH
Unimodal function										
F1	Best	300.02	30,224.81	2435.79	477.80	37,670.33	32,487.71	300.16	3008.42	**300**
	Mean	300.08	59,629.62	4778.73	2836.77	56,386.78	56,267.25	307.43	8776.13	**300**
	StD	0.145	9986.83	1631.82	2289.21	13,929.99	20,389.83	16.521	4062.34	**9.5 × 10**^**−12**^
Basic functions										
F2	Best	444.90	1504.50	449.28	415.00	463.19	912.12	414.82	428.68	**400**
	Mean	454.95	2200.73	465.98	462.83	513.93	1750.55	458.46	464.59	**439.81**
	StD	**10.311**	435.219	24.26	30.513	44.726	529.31	19.660	15.015	18.155
F3	Best	**600.24**	662.56	607.77	600.14	620.86	653.72	606.71	623.472	612.84
	Mean	**605.31**	684.74	629.01	602.34	639.25	684.30	629.32	645.34	634.42
	StD	4.190	7.393	9.322	**4.048**	7.358	12.220	10.058	9.661	11.915
F4	Best	**821.89**	968.55	835.56	849.58	829.52	922.31	853.73	845.77	842.78
	Mean	**850.46**	999.82	865.50	900.93	871.90	954.79	882.85	890.06	875.42
	StD	16.05	17.376	**15.214**	29.79	16.95	20.278	17.669	23.452	22.258
F5	Best	**930.94**	3277.01	992.25	1238.17	1666.064	2849.926	1249.81	1788.12	1206.33
	Mean	**1169.31**	4948.35	1513.81	2462.87	2371.00	3673.03	1848.52	2378.20	1874.44
	StD	**194.436**	859.562	376.653	636.69	375.57	471.658	343.371	304.611	324.049
Hybrid functions										
F6	Best	1882.54	90,365,085.95	2223.48	1947.71	25,861.92	57,613,430.85	1862.02	2022.830	**1800.30**
	Mean	4612.37	389,128,839.2	5328.58	13,826.44	155,894.16	867,615,884.2	5794.91	7461.50	**4610.99**
	StD	3201.410	190,973,416.5	3227.276	9392.515	117,944.76	838,420,299.2	4755.08	5751.65	**2683.249**
F7	Best	2027.05	2192.06	2052.78	**2023.81**	2070.94	2172.70	2035.89	2027.46	2069.44
	Mean	**2060.66**	2239.15	2110.38	2085.49	2133.50	2239.80	2117.42	2156.65	2142.50
	StD	26.909	**26.480**	38.913	52.669	72.288	46.489	40.241	66.119	55.245
F8	Best	2220.77	2286.92	2228.55	2221.19	2229.99	2238.83	2221.56	2228.03	**2210.52**
	Mean	**2224.82**	2418.70	2267.21	2253.21	2247.24	2422.96	2243.35	2238.87	2246.50
	StD	**6.695**	70.141	54.040	47.844	31.615	148.88	43.417	9.448	26.753
Composition functions										
F9	Best	2480.782	2720.715	2480.884	2480.782	2494.789	2639.303	2480.781	2480.782	**2480.781**
	Mean	2480.797	2913.051	2485.666	2486.190	2565.406	3000.960	2480.814	2483.454	**2480.781**
	StD	0.013	90.519	5.330	5.828	43.243	286.987	0.048	3.432	**3.63 × 10**^**−12**^
F10	Best	2500.472	2610.636	2500.668	**2414.572**	2500.670	5714.266	2500.754	2500.760	2500.745
	Mean	2878.745	2820.891	3040.516	**2806.015**	3065.910	6675.800	3726.354	3359.881	3745.876
	StD	639.116	**160.398**	855.475	165.485	910.325	514.280	1165.580	767.330	726.295
F11	Best	2900	5193.046	2619.646	2600.055	2876.708	5654.721	2900	**2600.002**	2900
	Mean	2930	6317.223	2941.369	2990.848	3186.476	7419.084	2965.112	**2910.003**	2916.667
	StD	46.609	539.848	167.852	161.029	185.885	816.256	88.733	95.952	**37.905**
F12	Best	2939.506	3158.671	2943.284	2940.273	2983.960	3213.355	2946.685	2940.228	**2938.921**
	Mean	2984.007	3266.632	2973.659	2967.016	3033.970	3559.546	3026.821	2981.706	**2972.224**
	StD	57.231	55.531	24.066	29.855	40.245	254.008	59.346	47.495	**30.449**
L1		3	0	0	2	0	0	0	1	**6**
L2		**5**	0	0	1	0	0	0	1	4
L3		3	2	1	1	0	0	0	0	**5**

The best results are marked in bold.

**Figure 24:**
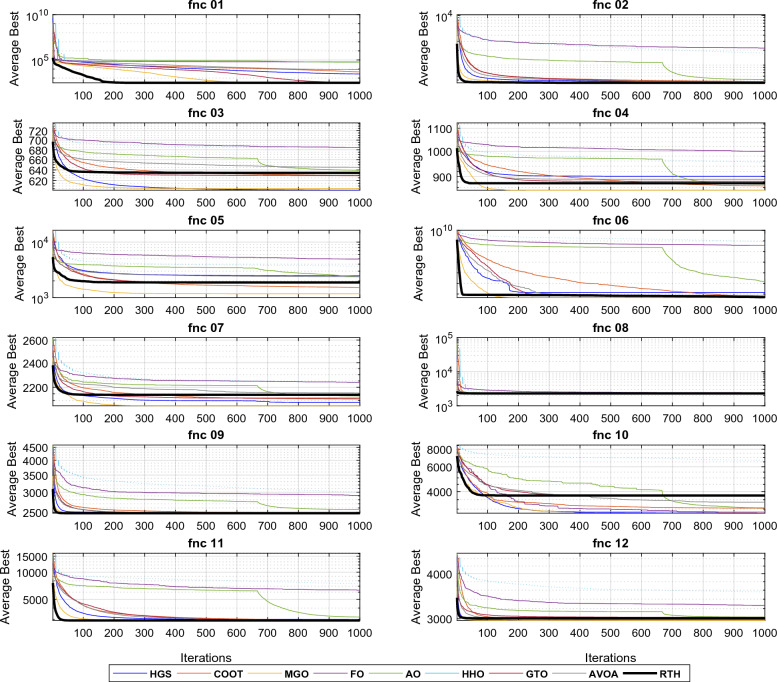
CEC 2022 fitness evolution for D = 10.

From the L1, the proposed RTH can find the optimal solution for six from twelve functions, followed by GTO by only three functions, then the HGS by two functions. The increased complexity can explain the decreased number of optimal solutions due to increased search space dimensions. These results approve the RTH's ability to get the optimal solution better and escape from the local solutions even in complicated problems. From L2, the MGO achieved the best mean results by five functions, followed by the proposed RTH by four. This approves the accuracy of the proposed RTH compared to the MGO algorithm. From L3, the proposed RTH provided the best results by five functions, followed by the proposed MGO by three times. This can approve its robustness. Compared to D = 10, the RTH can generally handle problems that include optimization variables better. Figure 24 presents the fitness evolution for each function of the CEC 2022 with D = 20. These figures approve the findings of the CEC 2020 and CEC 2022 with D = 10, where the proposed RTH presents a faster convergence speed.

**Figure 25:**
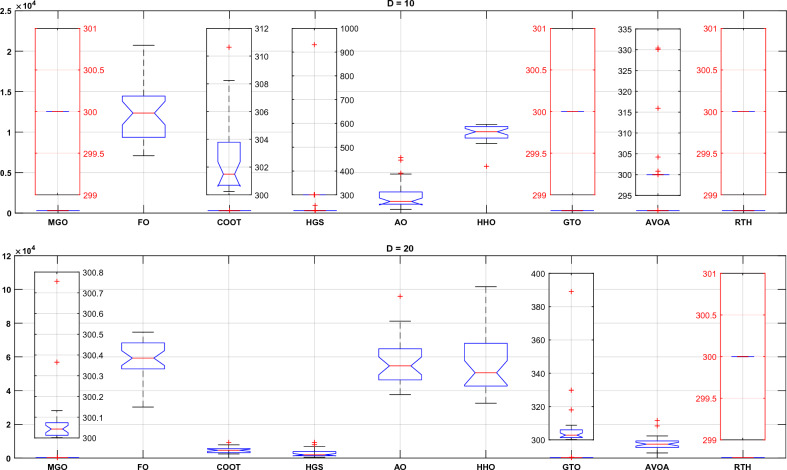
CEC 2022 unimodal function (F1) ANOVA results.

Figure 25 shows the ANOVA ranking results for unimodal functions for both cases. From this figure, the RTH and the MGO generate the best solutions in terms of accuracy with the lowest variation range for D = 10. However, when the search space dimensions increased, the performance of the MGO decreased where the RTH performance was constant.

**Figure 26:**
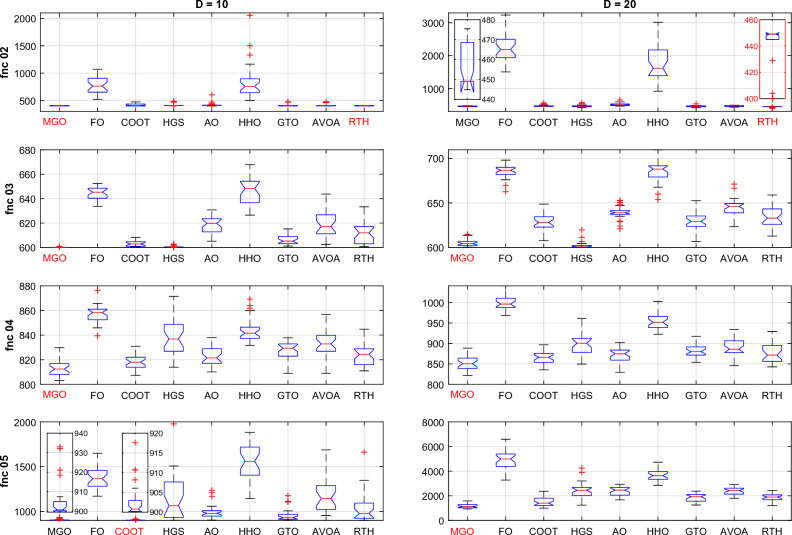
CEC 2022 basic functions ANOVA results.

The basic functions (fnc 02–05) results are presented in Fig. 26. For fnc 02 for both cases (D = 15 and D = 20), all the optimizers except for the FO and the HHO got the optimal solution where the MGO and RTH provided the lower variation range for both cases. The results of fnc 03 show that the proposed RTH cannot resolve this problem effectively. The MGO and the HGS provide the best performance for this case. For fnc 04, all the optimizers provided near performance with a slight advantage to the MGO. The same comments can be extracted for fnc 05.

**Figure 27:**
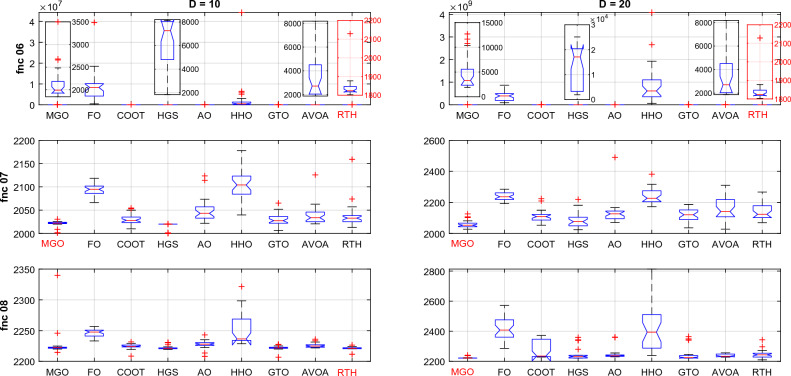
CEC 2022 hybrid functions ANOVA results.

Hybrid functions (fnc 06–08) ANOVA results are presented in Fig. 27. The results of the fnc 06 confirm the outperformance of the proposed RTH. All the optimizers provide similar performance for the other two functions with an advantage to the MGO, HGS, and RTH algorithms.

**Figure 28:**
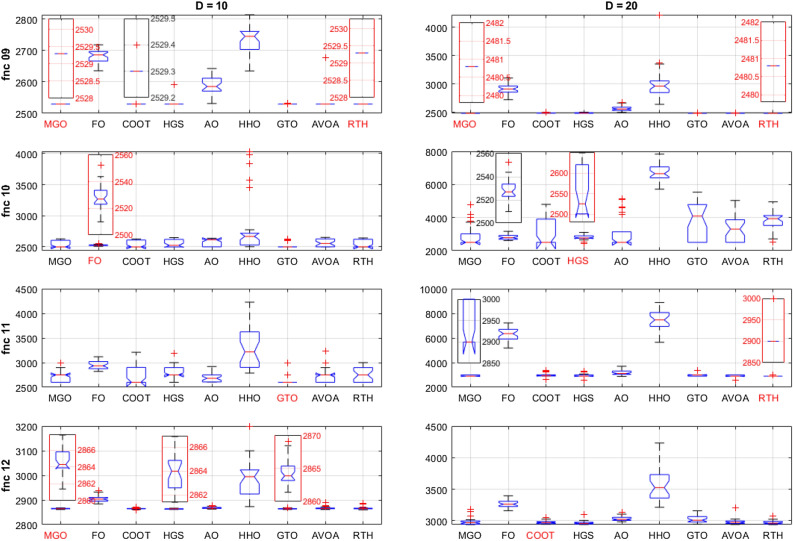
CEC 2022 composite functions ANOVA results.

Composition functions (fnc 09–12) ANOVA results are presented in Fig. 28. For function 09, the MGO and the RTH provided the best performance for both cases. For the other functions, the performance of the algorithms is much closer to each other, with light superiority for the FO and the HGS for fnc 10, the GTO and the RTH for fnc 11, and the MGO and the COOT for fnc 12.

**Figure 29:**
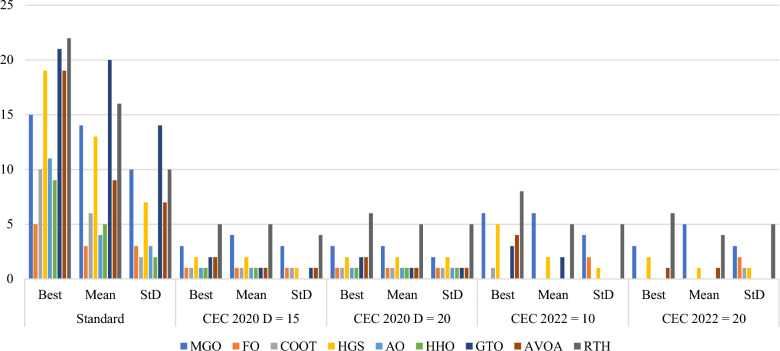
Classes one test results.

These mathematical test functions are used to elaborate the performance of the proposed RTH algorithm compared to other new and robust algorithms. The statistical results are summarized in Fig. 29.

From the results obtained, the RTH algorithm can provide very competitive performance for solving various optimization problems. The accuracy and stability of this proposed algorithm have been tested compared to the prementioned algorithms. The convergence speed has been checked and confirmed. After mathematically approving its performance, the proposed RTH algorithm will be tested with some published papers for several engineering tests.

## Engineering optimization problems

As mathematics and engineering real-world complex examples, the proposed RTH algorithm will be used to solve seven real-world complex engineering problems.

**Figure 30:**
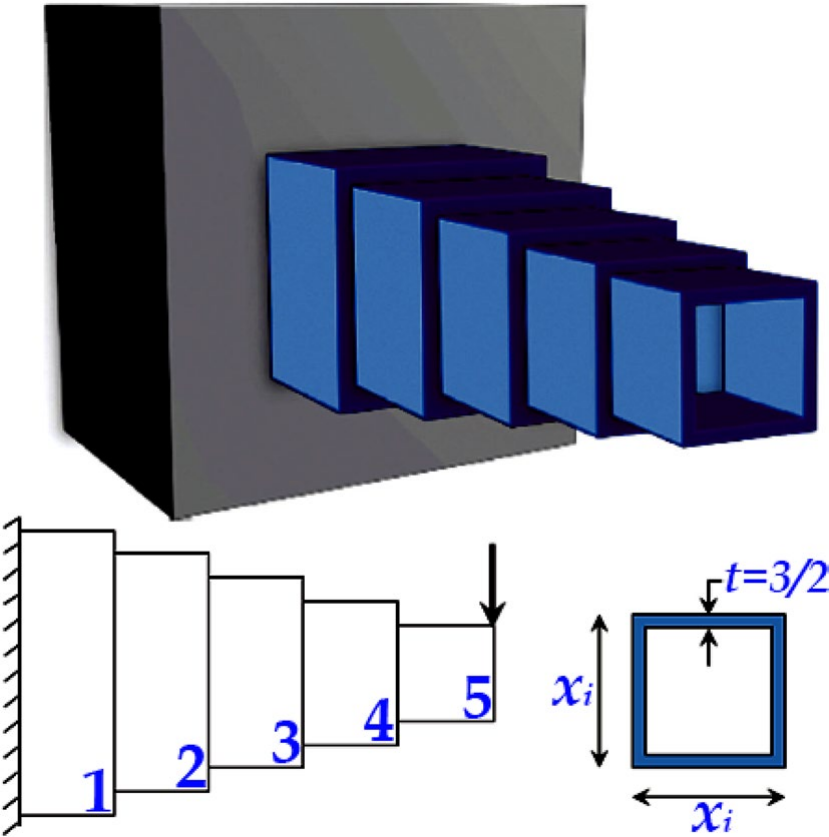
Cantilever beam schematic illustration.

- * Optimal design of I-Shaped beam*: this issue, which tries to reduce the vertical deflection of the beam, is another common engineering optimization problem^34^. It satisfies the cross-sectional area and stress restrictions simultaneously under specific loads, as explained in Fig. 30. This optimization problem can be expressed as follows:
  9 $$\begin{aligned} & f(x) = \frac{5000}{{x_{3} {\raise0.7ex\hbox{${\left( {x_{2} - 2x_{4} } \right)^{3} }$} \!\mathord{\left/ {\vphantom {{\left( {x_{2} - 2x_{4} } \right)^{3} } {12}}}\right.\kern-0pt} \!\lower0.7ex\hbox{${12}$}} + {\raise0.7ex\hbox{${x_{1} x_{4}^{3} }$} \!\mathord{\left/ {\vphantom {{x_{1} x_{4}^{3} } 6}}\right.\kern-0pt} \!\lower0.7ex\hbox{$6$}} + 2bx_{4} \left( {x_{2} - {\raise0.7ex\hbox{${x_{4} }$} \!\mathord{\left/ {\vphantom {{x_{4} } 2}}\right.\kern-0pt} \!\lower0.7ex\hbox{$2$}}} \right)^{2} }} \\ & g_{1} (x) = 2x_{1} x_{3} + x_{3} \left( {x_{2} - 2x_{4} } \right) \le 300 \\ & g_{2} (x) = \frac{{18x_{2} \times 10^{4} }}{{x_{3} \left( {x_{2} - 2x_{4} } \right)^{3} + 2x_{1} x_{3} \left( {4x_{4}^{2} + 3x_{2} \left( {x_{2} - 2x_{4} } \right)} \right)}} + \frac{{15x_{1} \times 10^{3} }}{{\left( {x_{2} - 2x_{4} } \right)x_{3}^{2} + 2x_{3} x_{1}^{3} }} \le 56 \\ & 10 \le x_{1} \le 50,\,\,\,10 \le x_{2} \le 80,\,\,\,0.9 \le x_{3} \le 5,\,\,\,0.9 \le x_{4} \le 5 \\ \end{aligned}$$
  where the optimization variables are: the width of the flange (*x*_*1*_), the height of the section (*x*_*2*_), the thickness of the web (*x*_*3*_), and the thickness of the flange (*x*_*4*_).

From Table 8, the proposed RTH obtains the best solutions for solving this optimization problem compared to other published algorithms. In addition, the PFOA provided similar performance to the proposed RTH, but the RTH achieved the stop criteria after 300 iterations, proving better convergence speed and accuracy.

**Figure 31:**
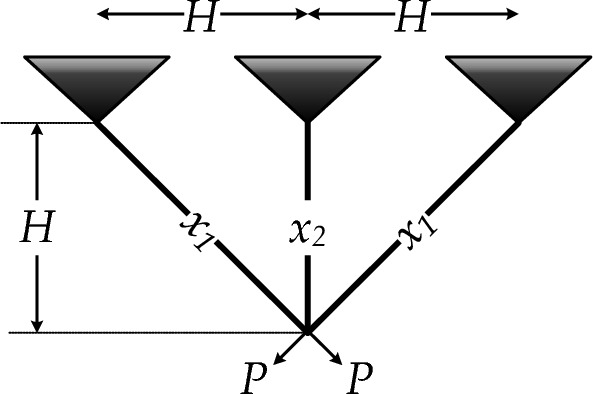
Three-bar truss schematic illustration.

(b) * Three-bar truss design*: In this example, a 3-bar planar truss is considered^34^, as represented in Fig. 31. The objective function and its constraints are presented as follows
  10 $$\begin{aligned} & f(x) = \left( {2\sqrt 2 x_{1} + x_{2} } \right) \times l \\ & g_{1} (x) = P\frac{{\sqrt 2 x_{1} + x_{2} }}{{\sqrt 2 x_{1}^{2} + 2x_{2} x_{1} }} - \sigma \le 0 \\ & g_{2} (x) = P\frac{{x_{2} }}{{\sqrt 2 x_{1}^{2} + 2x_{2} x_{1} }} - \sigma \le 0 \\ & g_{3} (x) = P\frac{{x_{2} }}{{\sqrt 2 x_{2} + x_{1} }} - \sigma \le 0 \\ & l = 100cm,\,\,P = 2{\raise0.7ex\hbox{${kN}$} \!\mathord{\left/ {\vphantom {{kN} {cm^{3} }}}\right.\kern-0pt} \!\lower0.7ex\hbox{${cm^{3} }$}},\,\,\,\sigma = 2{\raise0.7ex\hbox{${kN}$} \!\mathord{\left/ {\vphantom {{kN} {cm^{3} }}}\right.\kern-0pt} \!\lower0.7ex\hbox{${cm^{3} }$}},\,\,\,0 \le x_{1} ,\,\,\,x_{2} \le 1 \\ \end{aligned}$$

**Table 8:** Comparison of RTH results with the other algorithms for the I-shaped beam problem.

MA	Worst (× 10^−2^)	Mean (× 10^−2^)	Best (× 10^−2^)	StD(× 10^−5^)	Elapsed iterations
SOS^58^	NA	1.30884	1.30741	4.0	5000
CS^59^	1.35365	1.32165	1.30747	13.45	5000
AOS^60^	1.38140	1.31788	1.30741	15.55	100,000
SNS^34^	1.30764	1.30743	1.30741	4.31 × 10^−02^	3600
PFOA^61^	1.307412	1.307412	1.307412	2.05 × 10^−4^	24,000
AHA^62^	1.343036	1.340146	1.339957	7.91 × 10^−5^	15,000
FA^63^	1.339960	1.339957	1.339956	2.51 × 10^−7^	NA
RTH	1.307410	1.307410	1.307400	1.00 × 10^−8^	300

Table 9 provides the statistical results of these algorithms. It is clear that the best results are obtained using the proposed RTH algorithm and the AHA optimizer. However, the required number of iterations is only 670, much lower than the other reported algorithms, including the AHA, which requires 15,000 iterations to achieve the optimal value.

**Figure 32:**
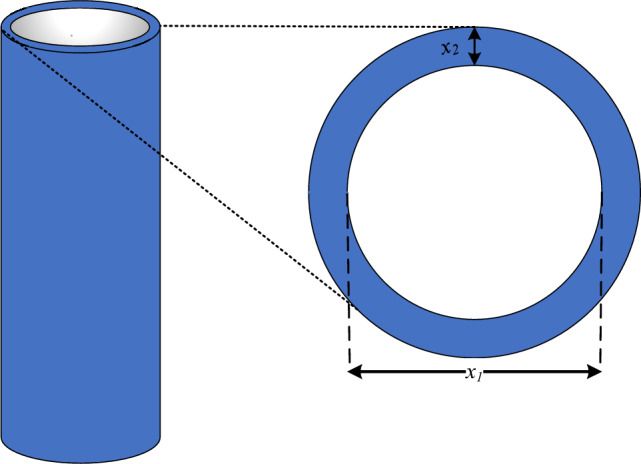
Tubular column schematic illustration.

(c) *Design of a tubular column*: this problem illustrates the structure of a uniform column with a tubular cross-section that can support a compressive load at the lowest possible cost^34^, as explained in Fig. 32. The two design variables in this issue are the mean diameter of the column (*x*_*1*_) and the thickness of the tube (*x*_*2*_). The column is constructed from a material having a yield stress of *y* and an elasticity modulus of *E*. The following equation represents this optimization problem:
  11 $$\begin{aligned} & f(x) = 9.8x_{1} x_{2} + 2x_{2} \\ & g_{1} (x) = \frac{P}{{\pi x_{2} x_{1} \sigma_{y} }} - 1 \le 0 \\ & g_{2} (x) = \frac{{8PL^{2} }}{{\pi^{3} Ex_{2} x_{1} \left( {x_{1}^{2} + x_{2}^{2} } \right)}} - 1 \le 0 \\ & g_{3} (x) = \frac{2}{{x_{1} }} - 1 \le 0,\,\,g_{4} (x) = \frac{{x_{1} }}{14} - 1 \le 0,\,\, \\ & g_{5} (x) = \frac{2}{{x_{2} }} - 1 \le 0,\,\,g_{6} (x) = \frac{{x_{2} }}{8} - 1 \le 0 \\ & \sigma_{y} = 500{\raise0.7ex\hbox{${kgf}$} \!\mathord{\left/ {\vphantom {{kgf} {cm^{2} }}}\right.\kern-0pt} \!\lower0.7ex\hbox{${cm^{2} }$}},\,\,P = 0.85 \times 10^{6} {\raise0.7ex\hbox{${kgf}$} \!\mathord{\left/ {\vphantom {{kgf} {cm^{3} }}}\right.\kern-0pt} \!\lower0.7ex\hbox{${cm^{3} }$}},\,\,\,\,\,2 \le x_{1} \le 14,\,\,\,0.2 \le x_{2} \le 0.8 \\ \end{aligned}$$

**Table 9:** Comparison of RTH results with the other used algorithms for the three-bar truss design problem.

MA	Worst	Mean	Best	StD	Elapsed iterations
CS^59^	NA	264.066900	263.9715600	9.00 × 10^−05^	15,000
GWO^64^	263.904218	263.897955	263.896006	1.61 × 10^−03^	50,000
WCA^65^	263.896201	263.895903	263.895843	8.71 × 10^−05^	5250
PSO^66^	264.584903	263.957414	263.895843	1.37 × 10^−01^	50,000
CGO^67^	263.896007	263.895851	263.895844	2.51 × 10^−05^	100,000
SNS^34^	263.895856	263.895846	263.895843	3.31 × 10^−6^	4800
PFOA^61^	263.895844	263.895843	263.895842	2.01 × 10^−6^	18,000
RL-BA^68^	263.924700	263.900300	263.895840	6.06 × 10^−6^	NA
AHA^62^	**263.895843**	**263.895843**	**263.895843**	1.09 × 10^−7^	15,000
RTH	**263.895843**	**263.895843**	**263.895843**	**5.78 × 10**^**−14**^	**670**

The best results are marked in bold.

This problem has already been tackled using several algorithms. The best results of these algorithms, as well as the suggested RTH, are shown in Table 10. Based on these results, the proposed RTH achieved the best results with the lowest number of iterations (300).

**Figure 33:**
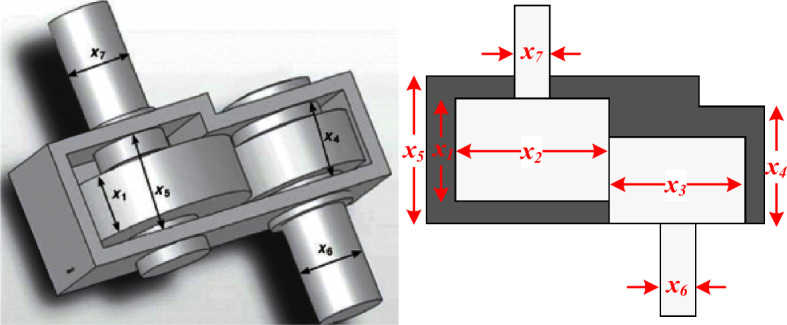
Speed reducer design schematic illustration.

(d) *Speed Reducer Design*: speed reducer is an essential part of the gearbox in mechanical systems. It may be used for a variety of purposes^71^. As shown in Fig. 33, the weight of the speed reducer must be reduced under 11 restrictions in this optimization problem. Face width (*x*_*1*_), the module of teeth (*x*_*2*_), number of teeth in the pinion (*x*_*3*_), length of the first shaft between bearings (*x*_*4*_), length of the second shaft between bearings (*x*_*5*_), the diameter of first shafts (*x*_*6*_), and diameter of the second shafts (*x*_*7*_) are the seven variables in this issue. This problem can be modeled as
  12 $$\begin{aligned} & f(x) = 0.7854x_{1} x_{2}^{2} \left( {3.3333x_{3}^{2} + 14.9334x_{3} - 43.0934} \right) \\ & - 1.508x_{1} \left( {x_{6}^{2} + x_{7}^{2} } \right) + 7.4777\left( {x_{6}^{3} + x_{7}^{3} } \right) + 0.7854\left( {x_{4} x_{6}^{2} + x_{5} x_{7}^{2} } \right) \\ & g_{1} (x) = \frac{27}{{x_{1} x_{3} x_{2}^{2} }} - 1 \le 0,\,\,\,g_{2} (x) = \frac{397.5}{{x_{1} x_{3} x_{2}^{2} }} - 1 \le 0 \\ & g_{3} (x) = \frac{1.93}{{x_{2} x_{3} x_{6}^{4} }} - 1 \le 0,\,\,\,g_{4} (x) = \frac{{1.93x_{5}^{3} }}{{x_{2} x_{3} x_{7}^{4} }} - 1 \le 0 \\ & g_{5} (x) = \frac{{\sqrt {\left( {{\raise0.7ex\hbox{${745x_{4} }$} \!\mathord{\left/ {\vphantom {{745x_{4} } {x_{2} x_{3} }}}\right.\kern-0pt} \!\lower0.7ex\hbox{${x_{2} x_{3} }$}}} \right)^{2} + 16.9 \times 10^{6} } }}{{110x_{6}^{3} }} - 1 \le 0 \\ & g_{6} (x) = \frac{{\sqrt {\left( {{\raise0.7ex\hbox{${745x_{5} }$} \!\mathord{\left/ {\vphantom {{745x_{5} } {x_{2} x_{3} }}}\right.\kern-0pt} \!\lower0.7ex\hbox{${x_{2} x_{3} }$}}} \right)^{2} + 157.5 \times 10^{6} } }}{{85x_{7}^{3} }} - 1 \le 0 \\ & g_{7} (x) = \frac{{x_{2} x_{3} }}{40} - 1 \le 0,\,\,\,g_{8} (x) = \frac{{5x_{2} }}{{x_{1} }} - 1 \le 0,\,\,\,g_{9} (x) = \frac{{x_{1} }}{{12x_{2} }} - 1 \le 0 \\ & g_{10} (x) = \frac{{1.5x_{6} + 1.9}}{{x_{4} }} - 1 \le 0,\,\,\,g_{9} (x) = \frac{{1.1x_{7} + 1.9}}{{x_{5} }} - 1 \le 0 \\ & 2.6 \le x_{1} \le 3.6,\,\,\,0.7 \le x_{2} \le 0.8,\,\,x_{3} \in \left[ {17,18, \cdots 28} \right],\,\,x_{4} \ge 7.3 \\ & x_{5} \le 8.3,\,\,\,2.9 \le x_{6} \le 3.9,\,\,\,5 \le x_{7} \le 5.5 \\ \end{aligned}$$

**Table 10:** RTH results and the other used algorithms for tubular column designing problem.

MA	Worst	Mean	Best	StD	Elapsed iterations
ISA^69^	26.532	26.531	26.531	1.70 × 10^−4^	3000
CS^59^	26.53972	26.53504	26.53217	1.93 × 10^−3^	15,000
AOS^60^	26.60831361	26.53161399	26.53137828	1.03 × 10^−3^	100,000
SNS^34^	26.48637095	26.48636249	**26.48636147**	2.22 × 10^−6^	1250
PFOA^61^	26.48636148	26.48636150	26.48636152	2.00 × 10^−8^	24,000
AOA^70^	27.16391	26.80510	26.53730	0.02761	3000
AOA-NM^70^	26.53169	26.53133	26.53132	5.36 × 10^−6^	3000
RTH	**26.48636147**	**26.48636147**	**26.48636147**	**7.23 × 10**^**−15**^	**300**

The best results are marked in bold.

The results of RTH and other published algorithms are reported in Table 11. Among these algorithms, the RTH has the lowest number of iterations, while its metrics in terms of best, mean, and StD are better than those other algorithms.

**Figure 34:**
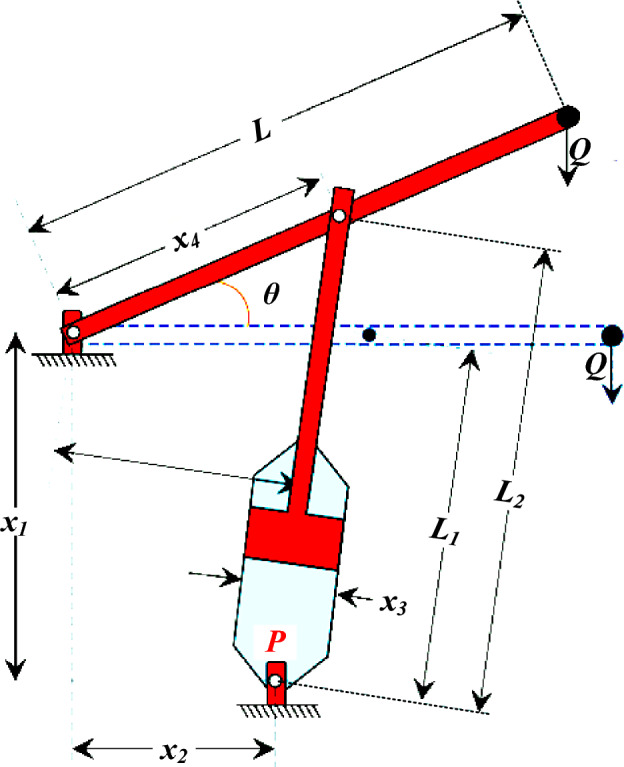
Piston lever design schematic illustration.

(e) *Piston lever*: the essential goal of this problem is to identify the piston components (*x*_*1*_), (*x*_*2*_), (*x*_*3*_), and (*x*_*4*_) by reducing the oil volume when the piston's lever is raised from 0° to 45°^34^. This problem is presented in Fig. 34***,*** and it can be modeled as follows:
  13 $$\begin{aligned} & f(x) = \frac{1}{4}\pi x_{3}^{2} \left( {L_{2} - L_{1} } \right) \\ & g_{1} (x) = QL\cos (\theta ) - RF \le 0 \\ & g_{2} (x) = Q\left( {L - x_{4} } \right) - M_{\max } \le 0 \\ & g_{3} (x) = 1.2\left( {L_{2} - L_{1} } \right) - L_{1} \le 0 \\ & g_{4} (x) = \frac{{x_{3} }}{2} - x_{2} \le 0 \\ & R = \frac{{ - x_{4} \left( {x_{4} \sin (\theta ) + x_{1} } \right) + x_{1} \left( {x_{2} - x_{4} \cos (\theta )} \right)}}{{L_{1} }} \\ & F = {\raise0.7ex\hbox{${\pi Px_{3}^{2} }$} \!\mathord{\left/ {\vphantom {{\pi Px_{3}^{2} } 4}}\right.\kern-0pt} \!\lower0.7ex\hbox{$4$}},\,\,L_{1} = \sqrt {(x_{4} - x_{2} )^{2} + x_{1}^{2} } ,\,\,\,L_{2} = \sqrt {\left( {x_{4} \sin (\theta ) + x_{1} } \right)^{2} + \left( {x_{2} - x_{4} \cos (\theta )} \right)^{2} } \\ & \theta = 45^\circ ,\,\,\,Q = 10000{\text{lbs}},\,\,L = 240{\text{in}},\,\,1.8 \times 10^{6} {\text{lbs}}{\text{.in,}}\,\,P = 1500{\text{psi}} \\ & 0.05 \le x_{1} \le 500,\,\,\,0.05 \le x_{2} \le 120 \\ \end{aligned}$$

**Table 11:** Comparison of RTH results with other algorithms for speed reducer designing problems.

MA	Worst	Mean	Best	StD	Elapsed iterations
CS^59^	3009.00000	3007.19970	3000.9810	4.96	250,000
ABC^72^	NA	2997.05841	2997.05841	0.20	30,000
WCA^65^	2994.50558	2994.47439	2994.47107	7.40 × 10^−3^	15,150
APSO^73^	4443.01764	3822.64062	3187.63047	3.66 × 10^−2^	30,000
CGO^67^	2995.50493	2994.46540	2994.44365	0.11	100,000
SNS^34^	2994.47110	2994.47101	2994.47107	7.00 × 10^−6^	3750
AHA^62^	2994.47116	2994.47165	2994.47323	4.25 × 10^−4^	30,000
ARSCA^74^	NA	NA	2995.821	NA	NA
RTH	2994.42400	2994.42400	2994.42400	4.63 × 10^−13^	212

According to the obtained results in Table 12, the SNS performs better than the proposed RTH algorithm in terms of mean and StD. However, achieving the stop criteria took many iterations (5000), whereas the RTH requires only 130 iterations.*f) Corrugated design*: This task seeks to reduce the weight of a corrugated bulkhead in a chemical tanker^66^. The optimization variables are width (*x*_*1*_), depth (*x*_*2*_), length (*x*_*3*_), and plate thickness (*x*_*4*_). Its mathematical model of this problem can be expressed as follows:

14 $$\begin{aligned} & f(x) = \frac{{5.885x_{4} \left( {x_{1} + x_{3} } \right)}}{{x_{1} + \sqrt {\left| {x_{3}^{2} - x_{2}^{2} } \right|} }} \\ & g_{1} (x) = - x_{4} x_{2} \left( {0.4x_{1} + \frac{{x_{3} }}{6}} \right) + 8.94\left( {x_{1} + \sqrt {\left| {x_{3}^{2} - x_{2}^{2} } \right|} } \right) \le 0 \\ & g_{2} (x) = - x_{4} x_{2}^{2} \left( {0.2x_{1} + \frac{{x_{3} }}{12}} \right) + 2.2\left( {8.94(x_{1} + \sqrt {\left| {x_{3}^{2} - x_{2}^{2} } \right|} )} \right)^{\frac{4}{3}} \le 0 \\ & g_{3} (x) = - x_{4} + 0.0156x_{1} + 0.015 \le 0 \\ & g_{4} (x) = - x_{4} + 0.0156x_{3} + 0.015 \le 0 \\ & g_{5} (x) = - x_{4} + 0.015 \le 0 \\ & g_{6} (x) = x_{2} - x_{3} \le 0 \\ & 0 \le x_{1} ,\,\,\,x_{2} \le 100,\,\,x_{3} \le 100,\,\,4 \le x_{4} \le 5, \\ \end{aligned}$$

**Table 12:** Comparison of RTH results with the other used algorithms for the piston lever problem.

MA	Worst	Mean	Best	StD	Elapsed iterations
CS^59^	168.5920	40.2319	8.4271	59.06	50,000
PSO^66^	294	166	122	51.7	50,000
ISA^69^	610.6	226.5	8.4	111.2	12,500
AOS^60^	167.6650	33.7413	8.4191	93.47	100,000
CGO^67^	167.4728	45.0487	8.4128	67.25	100,000
SNS^34^	167.4728	**24.3190**	8.4127	**47.72**	5000
SSA^75^	653.4973	276.9405	8.4220	121.42	25,000
MFO^75^	167.4727	91.1239	**8.4126**	80.27	25,000
MVO^75^	356.2368	138.4470	8.4289	138.51	25,000
EO^75^	167.4727	100.6675	8.4127	79.30	25,000
RTH	**167.4727**	30.8287	8.4127	71.54	**130**

The best results are marked in bold.

**Figure 35:**
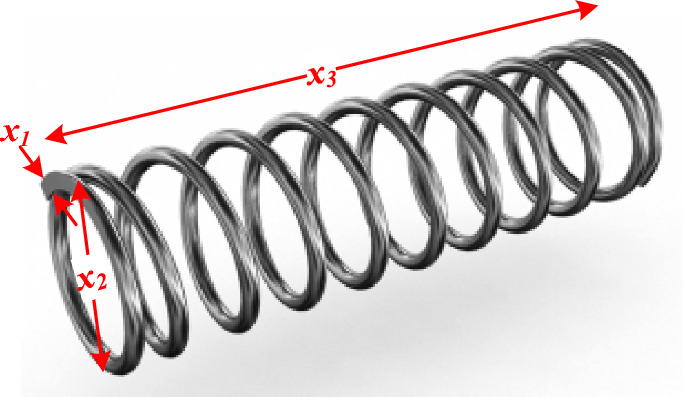
Speed reducer design schematic illustration.

The statistical results of this problem are provided in Table 13. Although AOA achieved the best results, these results were only obtained after 40,000 iterations, unlike the proposed RTH for the stop value after 129 iterations, which approves its solving speed.*g) Design of tension/compression spring*: tension/compression spring design challenge aims to decrease the weight of a tension/compression spring, as detailed in^78^ and Fig. 35. Minimum deflection, shear stress, surge frequency, outside diameter limitations, and design factors all play a role in this problem. The optimization variables include the mean coil diameter (*x*_*1*_), the wire diameter (*x*_*2*_), and the number of active coils (*x*_*3*_). This problem can be expressed as follows:

15 $$\begin{aligned} & f(x) = \left( {x_{3} + 2} \right)x_{2} x_{1}^{2} \\ & g_{1} (x) = 1 - \frac{{x_{3} x_{2}^{3} }}{{71785x_{1}^{4} }} \le 0 \\ & g_{2} (x) = \frac{{x_{3} x_{2}^{3} }}{{12566\left( {x_{2} x_{1}^{3} - x_{1}^{4} } \right)}} + \frac{1}{{5108x_{1}^{2} }} - 1 \le 0 \\ & g_{3} (x) = 1 - \frac{{140.45x_{1} }}{{x_{3} x_{2}^{2} }} \le 0,\,\,\,g_{4} (x) = \frac{{x_{1} + x_{2} }}{1.5} \le 0 \\ & 0.05 \le x_{1} \le 2,\,\,\,0.05 \le x_{1} \le 2,\,\,\,0.05 \le x_{1} \le 2,\,\,\, \\ \end{aligned}$$

**Table 13:** Comparison of RTH results with the other used algorithms for the corrugated bulkhead design problem.

MA	Worst	Mean	Best	StD	Elapsed iterations
FA^76^	NA	10.23	7.21	1.95	12,000
LF-FA^76^	NA	8.83	6.95	1.26	12,000
LS-LF-FA^76^	NA	7.44	6.86	0.67	12,000
AD-IFA^76^	NA	7.21	6.84	0.58	12,000
AOS^60^	7.06694	7.06081	6.84296	6.49 × 10^−04^	100,000
SNS^34^	6.84307	6.84298	6.84296	2.09 × 10^−05^	3125
DMO^77^	**5.9617**	**5.031**	**5.002**	0.996	40,000
RTH	6.84296	6.84296	6.84295	**4.51 × 10**^**−15**^	**129**

The best results are marked in bold.

Table 14 compares the RTH statistical results with other MAs. The RTH algorithm solves this problem in only 130 iterations, much less than the other considered algorithms with better results.

**Table 14:** Comparison of RTH results with the other used algorithms for tension/compression spring design problem.

MA	Worst	Mean	Best	StD	Elapsed iterations
PSO^66^	0.071802	0.019555	0.012857	1.17 × 10^−2^	20,000
CPSO^79^	0.012924	0.012730	0.012675	5.20 × 10^−5^	200,000
HPSO^80^	**0.012719**	0.012707	0.012665	1.58 × 10^−5^	81,000
WCA^65^	0.012952	0.012746	0.012665	8.06 × 10^−5^	11,750
MCEO^81^	0.013509	0.012720	**0.012661**	3.79 × 10^−5^	2000
EO^82^	0.013997	0.013017	0.012666	3.91 × 10^−4^	15,000
SNS^34^	0.012766	**0.012685**	0.012665	**2.39 × 10**^**−5**^	9000
SCMWOA^83^	NA	0.013400	0.012670	2.40 × 10^−4^	2460
RL-BA^68^	0.012928	0.012745	0.012676	7.19 × 10^−4^	NA
DDAO^84^	0.017320	0.0151829	0.012907	1.26 × 10^−3^	NA
RTH	0.013185	0.012776	0.012665	1.41 × 10^−4^	**300**

The best results are marked in bold.

## Proton Exchange membrane fuel cell parameters' identification

Fuel cells are recent energy-generation devices that produce efficient power by directly using fuel (mainly hydrogen) and oxygen while emitting no pollutants or noise (water, heat, and electricity). Because of its benefits and wide range of applications, proton exchange membrane fuel cells (PEMFCs) that employ polymers as electrolytes are becoming more prominent^85,86^. Fuel cell modeling is a crucial stage that enables the investigation and improvement of its performance^87^. The PEMFC model is built from nonlinear differential equations that explain internal chemical processes. These equations contain various empirical parameters that must be accurately identified to improve model accuracy. As a result, identifying these parameters is essential for creating an accurate model.

### PEMFC model

The PEMFC output voltage can be provided as follows^88^

16 $$V_{fc} = E_{Nernest} - V_{act} - V_{ohm} - V_{con}$$

where *V*_*act*_ is the activation voltage losses, *V*_*ohm*_ is the ohmic voltage losses, *V*_*con*_ represents the concentration voltage losses, and *E*_*Nernest*_ denotes the thermodynamic potential voltage (the Nernst voltage). *E*_*Nernest*_ can be calculated as follows:

17 $$E_{Nernest} = 1.229 - 0.85 \times 10^{ - 3} (T - 298.15) + 4.3085 \times 10^{ - 5} \times T\left( {\ln (P_{{H_{2} }} ) + \frac{{\ln (P_{{O_{2} }} )}}{2}} \right)$$

where *T* represents the operating temperature, *P*_*H2*_ and *P*_*O2*_ are the partial pressures of the hydrogen and the oxygen, respectively. They can be calculated as follows:

18 $$P_{{H_{2} }} = 0.5 \times R_{ha} \times P_{{H_{2} O}} \left[ {\frac{1}{{\frac{{R_{ha} \times P_{{H_{2} O}} }}{{P_{a} }} \times e^{{\frac{{1.635\left( {{i \mathord{\left/ {\vphantom {i A}} \right. \kern-0pt} A}} \right)}}{{T^{1.334} }}}} }} - 1} \right]$$

19 $$P_{{H_{2} }} = 0.5 \times R_{ha} \times P_{{H_{2} O}} \left[ {\frac{1}{{\frac{{R_{ha} \times P_{{H_{2} O}} }}{{P_{a} }} \times e^{{\frac{{1.635\left( {{i \mathord{\left/ {\vphantom {i A}} \right. \kern-0pt} A}} \right)}}{{T^{1.334} }}}} }} - 1} \right]$$

where *R*_*ha*_ and *R*_*hc*_ represent the vapor humilities of both anode and cathode, *P*_*a*_ and *P*_*c*_ present the inlet pressures of both anode and cathode (atm), *A* is the electrode surface (cm^2^), *i* is the current of the FC (A), and *P*_*H2O*_ represents the water vapor saturation pressure(atm).

The activation voltage losses (*V*_*act*_) can be obtained as

20 $$V_{act} = - \left( {\zeta_{1} + \zeta_{2} T + \zeta_{3} T\ln (C_{{O_{\partial } }} ) + \zeta_{4} T\ln (i)} \right)$$

where ξ_1_, ξ_2_, ξ_3_, ξ_4_ denote semi-empirical parameters; C_O2_ is the concentration of oxygen at the cathode's surface (mol.cm^−3^). It can be calculated as follows:

21 $$C_{{O_{2} }} = \frac{{P_{{O_{2} }} }}{{5.08 \times 10^{6} }}e^{{^{{\left( \frac{498}{T} \right)}} }}$$

The ohmic losses (*V*_*ohm*_) can be obtained as

22 $$V_{ohm} = i(R_{m} + R_{c} )$$

where *R*_*c*_ is the resistance of the connectors, and *R*_*m*_ is the resistance of the membrane. *R*_*m*_ can be calculated as

23 $$R_{m} = \rho_{m} \left( {\frac{l}{{A_{m} }}} \right)$$

where *l* and *A*_*m*_ represent the membrane thickness (cm) and surface (cm^2^), respectively, and *ρ*_*m*_ represents the membrane-specific resistivity (ohm × cm). *ρ*_*m*_ can be obtained as

24 $$\rho_{m} = \frac{{181.6\left[ {1 + 0.03{\raise0.7ex\hbox{$i$} \!\mathord{\left/ {\vphantom {i {A_{m} }}}\right.\kern-0pt} \!\lower0.7ex\hbox{${A_{m} }$}} + 0.062{\raise0.7ex\hbox{$T$} \!\mathord{\left/ {\vphantom {T {303}}}\right.\kern-0pt} \!\lower0.7ex\hbox{${303}$}}\left( {{\raise0.7ex\hbox{$i$} \!\mathord{\left/ {\vphantom {i {A_{m} }}}\right.\kern-0pt} \!\lower0.7ex\hbox{${A_{m} }$}}} \right)^{2.5} } \right]}}{{\left[ {\lambda - 0.634 - 3{\raise0.7ex\hbox{$i$} \!\mathord{\left/ {\vphantom {i {A_{m} }}}\right.\kern-0pt} \!\lower0.7ex\hbox{${A_{m} }$}}} \right]e^{{4.18\frac{T - 303}{T}}} }}$$

where *λ* is the membrane material's water content.

### Objective function

The sum square error (SSE) between the measured (*V*_data_) and model output data (*V*_data_) will be used as an objective function. The objective function can be constructed as follows:

25 $$f(x) = \sum\nolimits_{k = 1}^{N} {(V_{data} (k) - V_{model} (k,x))^{2} }$$

where *N* represents the data size, and *x* represents a vector containing seven unknown parameters.

26 $$x = \left[ {\begin{array}{*{20}c} {\zeta_{1} } & {\zeta_{2} } & {\zeta_{3} } & {\zeta_{4} } & {R_{c} } & {\begin{array}{*{20}c} \lambda & b \\ \end{array} } \\ \end{array} } \right]$$

The FC data are compared with those generated by the model, and the fitness value is calculated based on the error between them. The model is developed in MATLAB script, and the data are loaded from the Excel sheet file. The identification is an iterative process that updates the candidate solutions at each iteration by sending them to the MATLAB script that includes the FC model and simulates it after that, and then generates the fitness value. This process repeated until the last iteration.

### Results

The suggested RTH algorithm will be used to extract the seven unknown characteristics of three PEM fuel cells: the NedStack PS6, the BCS 500W, and the SR-12 500W. Table 15^87^ provided the accurate values of the parameters, testing operating conditions, and measurement data for the tested PEMFC types. The upper and lower limits of the empirical parameters are presented in Table 16. The results are compared to those published for the same FC types.

**Table 15:** The characteristics of the considered PEMFCs.

	NedStack PS6	BCS 500W	SR-12 500W
*N* (number of cells)	65	32	32
*A* (cm^2^)	240	64	64
*l* (μm)	178	178	178
$P_{H2}^{*}$(bar)	1.0	1.0	1
$P_{O2}^{*}$(bar)	1.0	1.0	0.2095
*T* (K)	343	333	333
*RH*_*a*_	100%		
*RH*_*c*_	100%

**Table 16:** The upper and the lower limits of the empirical parameters.

Optimization variables	$\xi_{1}$	$\xi_{2}$	$\xi_{3}$	$\xi_{4}$	$\lambda$	$R_{C} \left(\Omega \right)$	*b* (V)
Lower limit	− 1.19969	0.001	$3.6 \times 10^{ - 5}$	${-}2.6 \times 10^{ - 4}$	10	$1 \times 10^{ - 4}$	0.0136
Upper limit	− 0.8532	0.005	$9.8 \times 10^{ - 5}$	${-}9.54 \times 10^{ - 5}$	24	$8 \times 10^{ - 4}$	0.5

Table 17 shows the comparative findings for the BCS 500 W type, Table 18 for the NedStack PS6, and Table 19 for the SR-12 PEM 500 W. The comparison findings are primarily based on sum square error (SSE).

**Table 17:** The extracted BSC 500W FC parameters.

Parameters	SSA^89^	PO^90^	MPA^90^	IAEO^91^	MAEO^92^	ISSA^93^	EHBO^94^	HGSA^95^	NNA^96^	RTH
$\xi_{1}$	− 1.010	− 1.200	− 0.986	− 0.810	− 0.856	− 1.098	− 1.200	− 1.11	− 1.060	− 1.029
$\xi_{2}$ × 10^−3^	3.220	4.042	2.609	5.17	2.73328	3.3352	3.310	3.753	3.744	3.100
$\xi_{3}$ × 10^−5^	5.450	9.800	3.600	8.790	6.634	5.903	4.200	9.710	9.690	6.487
$\xi_{4}$. × 10^−5^	− 1.420	− 1.929	− 1.929	− 1.900	− 1.928	− 1.928	− 1.930	− 1.935	− 19.302	− 1.936
${\uplambda }$	20.710	20.818	20.817	20.877	20.703	21.250	20.877	21.970	20.877	22.02
$R_{C} \left(\Omega \right)$ × 10^−4^	0.075	0.016	0.016	0.161	0.100	0.161	0.100	0.100	0. 100	0.100
*b* (V) × 10^−2^	1.00	1	1	1.00	1.60	148.2	1.60	1.60	1.60	1.740
Best	1.219	1.156	1.156	1.16	1.157	1.16	1.170	1.169	1.1698	**1.140**
Worst	1.520	NA	NA	1.17	NA	179.26	1.185	1.34	1.3670	**1.140**
Mean	NA	1.88	1.16	1.16	2.9326	14.57	1.174	NA	NA	**1.140**
StD	871 × 10^−2^	NA	NA	144 × 10^−3^	NA	NA	0.6 × 10^−4^	3. 4 × 10^−4^	5.64 × 10^−4^	**9.37 × 10**^**−8**^

The best results are marked in bold.

**Table 18:** The extracted NedStack PS6 FC parameters.

	SSA^89^	EO^97^	STSA^98^	IAEO^91^	EBHO^94^	mAEFA^99^	NNA^96^	RTH
ξ_1_	1.130	− 1.12171	− 0.853	− 1.1997	− 0.85396	− 1.149	− 0.8535	− 0.90568
ξ_2_ × 10^−5^	3.460	3.77	2.840	3.4103	2.40	3.349	2.4316	3.44
ξ_3_ × 10^−5^	4.590	7.81	6.790	3.60	3.60	3.60	3.7545	8.76
ξ_4_ × 10^−5^	− 9.620	− 9.54	− 9.540	− 9.54	− 9.54	− 9.5	− 9.5400	− 9
*λ*	12.910	16.60171	13.463	19.7903	13.465	13.097512	13.0802	17.80574
*R*_*c*_ × 10^−3^	0.100	0.205	0.100	0.362	0.1	0.1	0.1000	8
*b* × 10^−2^	6.000	0.0285	1.360	1.360	1.360	1.360	1.360	8.185
Best	2.181	2.40931	2.146	2.1459	2.14570	2.07974	2.14487	**2.1058**
Worst	2.251	3.02680	3.183	2.1459	2.14570	2.08019	2.1645	**2.1058**
Mean	NA	2.61680	0.280	2.1459	2.14570	2.07987	NA	**2.1058**
StD	0.020	0.148	0.177	NA	5.69 × 10^3^	1.6 × 10^−4^	5.848 × 10^3^	**1.79 × 10**^**−6**^

The best results are marked in bold.

**Table 19:** The extracted SR-12 500W FC parameters.

Parameters	PO^90^	LSHADE^100^	MPA^90^	VSDE^101^	MAEO^92^	ISSA^93^	TGA^102^	RTH
$\xi_{1}$	− 0.860	1.216	− 1.028	− 0.952	− 0.860	− 159	− 1.112	− 0.906
$\xi_{2}$ × 10^−3^	3.376	− 0.960	3.898	3.000	2.771	4.146	3.855	3.440
$\xi_{3}$ × 10^−5^	9.794	2.621	9.800	7.783	6.170	5.6443	4.370	8.760
$\xi_{4}$. × 10^−5^	− 0.954	3.60	− 0.954	− 2.00	− 0.954	− 2.2908	− 0.964	− 9.00
${\uplambda }$	23.00	− 9.54	23.00	20.29	22.99	13.78	23.00	17.806
${{R}}_{{{C}}} \left(\Omega \right)$ × 10^−4^	6.723	0.154	6.723	1.00	6.707	1.00	2.19	8.00
*b* (V) × 10^−2^	17.5	23.999	17.5	2.79	17.5	7.4	18.3	8.185
Best	1.057	1.216	1.057	1.0526	1.057	0.792	1. 104	**0.5607**
Worst	NA	3.508	NA	1.1875	NA	1.793	5.504	**0.5607**
Mean	1.058	3.001	1.057	1.0834	6.43	1.46	2.064	**0.5607**
StD	NA	0.12402	NA	0.1768	NA	NA	NA	**1.79 × 10**^**−6**^

The best results are marked in bold.

**Figure 36:**
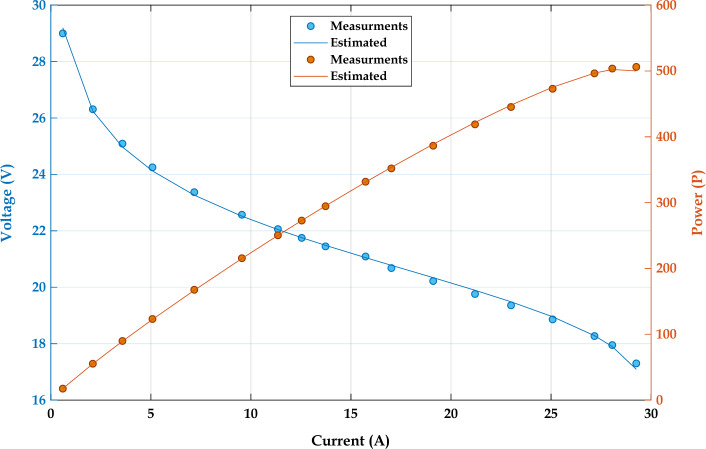
Voltage and power curves of BCS 500W using the proposed RTH algorithm.

Based on the findings reported in Table 17, the suggested method-based RTH performed the best of all the reported techniques in this comparison to extract the parameters of the BCS 500W. The proposed RTH algorithms obtained the minimal fitness function (SSE) by 1.14 × 10^−2^. On the other hand, the mean fitness values were similar to the best value. The RTH provides the lowest STD values compared to the different algorithms (9.37 × 10^−8^), indicating its robustness. Figure 36 depicts the experimental and estimated voltage and power curves of BCS 500W using the suggested RTH algorithm. The estimated voltage and power curves match the experimental curves. These curves demonstrate the proposed algorithm's accuracy in deriving the best BCS 500W parameters.

**Figure 37:**
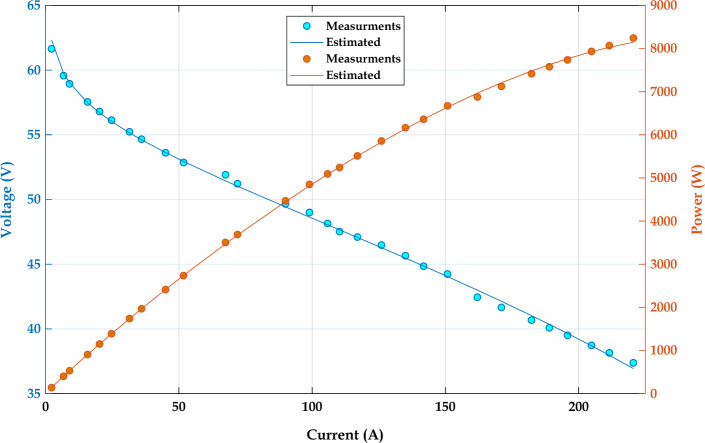
Voltage and power curves of BCS 500W using the proposed RTH algorithm.

Similar to the BDC 500W case, the suggested method-based RTH performed the best of all the cited methods in this comparison to extract the parameters of the NedStack PS6. The proposed RTH best result is 2.058, similar to the mean values. The RTH provides the lowest StD values compared to the different algorithms (1.79 × 10^−6^), approving its robustness. Figure 37 depicts the experimental and estimated voltage and power curves of NedStack PS6 using the suggested RTH algorithm. The estimated voltage and power curves match the experimental curves.

**Figure 38:**
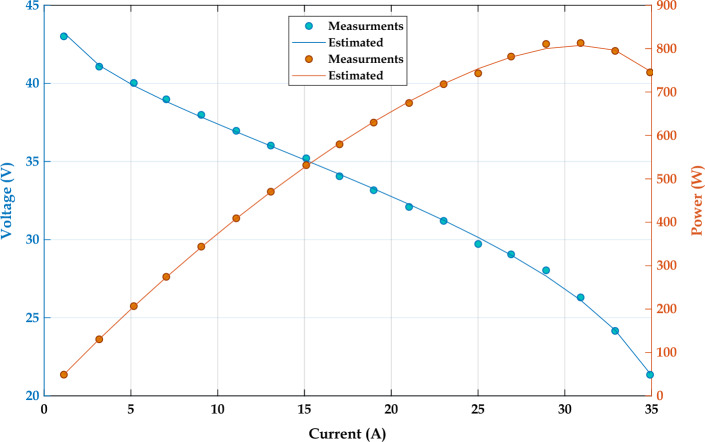
Voltage and power curves of SR-12 500W using the proposed RTH algorithm.

Similar to the previous cases, the suggested method-based RTH performed the best of all the cited methods in this comparison to extract the parameters of the SR 500W. The proposed RTH best result is 0.5607, similar to the mean values. The RTH also provides the lowest StD value by 1.79 × 10^−6^, approving its robustness. Figure 38 shwos the experimental and estimated voltage and power curves of SR 500W using the suggested RTH algorithm.

The obtained results for the standard test functions evaluated the performance of the proposed RTH algorithm for both exploitation and exploitation phases compared to other MAs. From these results, the proposed RTH has excellent exploitation and exploration abilities. In addition, its convergence speed has been approved. Then, the proposed RTH efficiently has been evaluated for several real-world applications. Its performance has been compared to several published algorithms for each problem. The achieved results confirm its ability to handle these problems. Finally, a PEMFC parameters extraction has been performed using the proposed RTH algorithm. The results have been compared to other well-known algorithms to approve its performance. The results achieved were excellent. Hence, its performance has been elaborated and approved for various types of optimization problems.

## Conclusion and future works

This paper has proposed a novel metaheuristic optimization algorithm named the red-tailed hawk (RTH) algorithm to solve various optimization tasks and problems. The proposed RTH is inspired by the red-tailed hawk's hinting behaviors of a predatory bird. A mathematical model has been developed to replicate the behavior of red-tailed hawks. The main contribution of this paper is to propose a new optimizer that has high robustness and fast convergence speed when solving various optimization problems. RTH's performance was firstly evaluated using three types of mathematical functions that express the nature of different optimization problems: twenty-three standard benchmark test functions, IEEE Congress on Evolutionary Computation 2020 (CEC2020) with 15 and 20 search space dimensions, and CEC2022 with 10 and 20 search space dimensions. These functions enable evaluating the exploitative ability, exploratory ability, and local optima avoidance of RTH. The results are compared to other recent and robust optimizers, including Farmland Fertility Optimizer (FO), African Vultures Optimization Algorithm (AVOA), Mountain Gazelle Optimizer (MGO), Gorilla Troops Optimizer (GTO), COOT algorithm, Hunger Games Search (HGS), Aquila Optimizer (AO), and Harris Hawks optimization (HHO). The results show that the proposed algorithm can provide the optimal solution for most of the considered functions with fast convergence speed and good robustness. Then, the findings of the seven constrained engineering design problems demonstrated that the RTH could show superior results to other published algorithms in terms of precision, robustness, and convergence rate. To deeply investigate the performance of the proposed RTH, the results of the proton exchange membrane fuel cell parameters extractions (PEMFC) have used the proposed RTH algorithm compared to published ones. The ultimate results show the RTH's ability to find better parameters for the dynamic model of the PEMFC.

RTH’s performance is anticipated to be considerably improved by integrating more complicated processes and combining effective operators and technics of other heuristics. Enhancing the proposed algorithm by including other factors in the model, such as the wind effect and prey escaping, is possible. However, the bigger code size of the RTH compared to other algorism like the PSO and the SSA can be a problem for its implementation. Bus, this problem can be bypassed with the utilization of fast calculators.

### Supplementary Information

Supplementary Tables.

Supplementary Information 2.

Supplementary Information 3.

Supplementary Information 4.

Supplementary Information 5.

Supplementary Information 6.

Supplementary Information 7.

Supplementary Information 8.

## Data Availability

All data generated or analyzed during this study are included in this published article and its supplementary information files.
