# Supplementary material for: Red-tailed hawk algorithm for numerical optimization and real-world problems
Source: Sci Rep. 2023 Aug 9;13:12950. doi: 10.1038/s41598-023-38778-3 (PMC10412609; doi:10.1038/s41598-023-38778-3)
Supplement: Supplementary file 1 — Supplementary Tables. [file 41598_2023_38778_MOESM1_ESM.docx]

**Appendix**

**TableA1:** standard test functions

| **Function number** | **Test function** | **Search space range** | **dim** |
| --- | --- | --- | --- |
| **Unimodal functions** | | | |
| F1 | Sphere | [− 100, 100] | 10 |
| F2 | Schwefel 2.22 | [− 10, 10] | 10 |
| F3 | Schwefel 1.2 | [− 100, 100] | 10 |
| F4 | Schwefel 2.21 | [− 100, 100] | 10 |
| F5 | Rosenbrock | [− 30, 30] | 10 |
| F6 | Step | [− 100, 100] | 10 |
| F7 | Quartic | [− 1.28, 1.28] | 10 |
| **Multimodal functions** | | | |
| F8 | Schwefel | [− 500, 500] | 10 |
| F9 | Rastrigin | [− 5.12, 5.12] | 10 |
| F10 | Ackley | [− 32, 32] | 10 |
| F11 | Griewank | [− 600, 600] | 10 |
| F12 | Penalized | [− 50, 50] | 10 |
| F13 | Penalized 2 | [− 50, 50] | 10 |
| **Fixed-dimensional multimodal function** | | | |
| F14 | Foxholes | [− 65.536, 65.536] | 2 |
| F15 | Kowalik | [− 5, 5] | 4 |
| F16 | Six Hump Camel | [− 5, 5] | 2 |
| F17 | GoldStein-Price | [− 2, 2] | 2 |
| F18 | Hartman 3 | [0, 1] | 3 |
| F19 | Hartman 6 | [0, 1] | 6 |
| F20 | Shekel 5 | [0, 10] | 4 |
| F21 | Shekel 7 | [0, 10] | 4 |
| F22 | Shekel 10 | [0, 10] | 4 |

**TableA2:** List of the considered CEC 2020 benchmark functions

| Number | Description | Lb | Ub | Solution |
| --- | --- | --- | --- | --- |
|  | ***Unimodal function*** | | | |
| F1 | Shifted and Rotated bent cigar function | -100 | 100 | 100 |
|  | ***Basic functions*** | | | |
| F2 | Shifted and rotated Schwefel's function | -100 | 100 | 1100 |
| F3 | Shifted and rotated Lunacek bi-Rastrigin function | -100 | 100 | 700 |
| F4 | Expanded Rosenbrock's plus Griewangk's function | -100 | 100 | 1900 |
|  | ***Hybrid functions*** | | | |
| F5 | Hybrid function 1 (N = 3) | -100 | 100 | 1700 |
| F6 | Hybrid function 2 (N = 4) | -100 | 100 | 1600 |
| F7 | Hybrid function 3 (N = 5) | -100 | 100 | 2100 |
|  | ***Composition functions*** | | | |
| F8 | Composition function 1 (N = 3) | -100 | 100 | 2200 |
| F9 | Composition function 1 (N = 3) | -100 | 100 | 2400 |
| F10 | Composition function 1 (N = 3) | -100 | 100 | 2500 |

**TableA3:** List of the considered CEC 2022 benchmark functions

| Number | Description | Lb | Ub | Solution |
| --- | --- | --- | --- | --- |
|  | ***Unimodal function*** | | | |
| F1 | Shifted and Rotated bent cigar function | -100 | 100 | 300 |
|  | ***Basic functions*** | | | |
| F2 | Shifted and full Rotated Rosenbrock's Function | -100 | 100 | 400 |
| F3 | Shifted and full Rotated Expanded Schaffer's f6 Function | -100 | 100 | 600 |
| F4 | Shifted and full Rotated Non-Continuous Rastrigin's Function | -100 | 100 | 800 |
| F5 | Shifted and full Rotated Levy Function | -100 | 100 | 900 |
|  | ***Hybrid functions*** | | | |
| F6 | Hybrid function 1 (N = 3) | -100 | 100 | 1800 |
| F7 | Hybrid function 2 (N = 6) | -100 | 100 | 2000 |
| F8 | Hybrid function 3 (N = 5) | -100 | 100 | 2200 |
|  |  |  |  |  |
|  | ***Composition functions*** | | | |
| F9 | Composition function 1 (N = 5) | -100 | 100 | 2300 |
| F10 | Composition function 2 (N = 4) | -100 | 100 | 2400 |
| F11 | Composition function 3 (N = 5) | -100 | 100 | 2600 |
| F12 | Composition function 4 (N = 6) | -100 | 100 | 2700 |
